# A tailored intervention to promote uptake of retinal screening among young adults with type 2 diabetes - an intervention mapping approach

**DOI:** 10.1186/s12913-018-3188-5

**Published:** 2018-05-31

**Authors:** Amelia J. Lake, Jessica L. Browne, Charles Abraham, Dee Tumino, Carolyn Hines, Gwyneth Rees, Jane Speight

**Affiliations:** 1 School of Psychology, Deakin University, Geelong, Australia; 2 The Australian Centre for Behavioural Research in Diabetes, Diabetes Victoria, Melbourne, Australia; 3 Institute of Health Research, University of Exeter Medical School, Exeter, UK; 4 Vision 2020 Australia, Melbourne, Australia; 5 Diabetes Victoria, Melbourne, Australia; 6 Centre for Eye Research Australia, Royal Victorian Eye and Ear Hospital, Melbourne, 3002 Australia; 7 Ophthalmology, Department of Surgery, University of Melbourne, Melbourne, 3010 Australia; 8 AHP Research, Hornchurch, UK

**Keywords:** Type 2 diabetes, Young adults, Diabetic retinopathy, Intervention mapping, Needs assessment, Retinal screening, Young-onset, Health behaviour change

## Abstract

**Background:**

Young adults (18–39 years) with type 2 diabetes are at risk of early development and rapid progression of diabetic retinopathy, a leading cause of vision loss and blindness in working-age adults. Retinal screening is key to the early detection of diabetic retinopathy, with risk of vision loss significantly reduced by timely treatment thereafter. Despite this, retinal screening rates are low among this at-risk group. The objective of this study was to develop a theoretically-grounded, evidence-based retinal screening promotion leaflet, tailored to young adults with type 2 diabetes.

**Methods:**

Utilising the six steps of Intervention Mapping, our multidisciplinary planning team conducted a mixed-methods needs assessment (Step 1); identified modifiable behavioural determinants of screening behaviour and constructed a matrix of change objectives (Step 2); designed, reviewed and debriefed leaflet content with stakeholders (Steps 3 and 4); and developed program implementation and evaluation plans (Steps 5 and 6).

**Results:**

Step 1 included in-depth qualitative interviews (*N* = 10) and an online survey that recruited a nationally-representative sample (*N* = 227), both informed by literature review. The needs assessment highlighted the crucial roles of knowledge (about diabetic retinopathy and screening), perception of personal risk, awareness of the approval of significant others and engagement with healthcare team, on retinal screening intentions and uptake. In Step 2, we selected five modifiable behavioural determinants to be targeted: knowledge, attitudes, normative beliefs, intention, and behavioural skills. In Steps 3 and 4, the “Who is looking after your eyes?” leaflet was developed, containing persuasive messages targeting each determinant and utilising engaging, cohort-appropriate imagery. In Steps 5 and 6, we planned Statewide implementation and designed a randomised controlled trial to evaluate the leaflet.

**Conclusions:**

This research provides an example of a systematic, evidence-based approach to the development of a simple health intervention designed to promote uptake of screening in accordance with national guidelines. The methods and findings illustrate how Intervention Mapping can be employed to develop tailored retinal screening promotion materials for specific priority populations. This paper has implications for future program planners and is intended to assist those wishing to use Intervention Mapping to create similar theoretically-driven, tailored resources.

**Electronic supplementary material:**

The online version of this article (10.1186/s12913-018-3188-5) contains supplementary material, which is available to authorized users.

## Background

Worldwide increase in the prevalence of type 2 diabetes (T2D) in young adults (< 40 years), with its associated considerable morbidity and mortality, is a burgeoning public health concern [1–5]. Adverse phenotype [6], sub-optimal glycemic (blood glucose) control and long diabetes duration expose young adults with T2D to a high lifetime risk of diabetes-related complications [7, 8]. One of the most common is diabetic retinopathy (DR), which is a leading cause of vision loss and blindness in working age adults [9, 10].

Early detection of DR via retinal screening (‘screening’), followed by timely treatment, are crucial factors in preventing vision loss [11]. Australian national Guidelines for the Management of Diabetic Retinopathy recommend screening uptake at diabetes diagnosis, repeated at least every two years thereafter [12], an interval less frequent than that prescribed for adults with T2D in the United States (US) and United Kingdom (UK) [13, 14]. Unfortunately however, young adults (aged 18–39 years) are the least likely to initiate retinal screening in accordance with national guidelines and have lower overall screening rates than older adults (aged ≥40 years) or young adults with type 1 diabetes [15–17]. In addition to their low engagement with existing diabetes services [18], additional communication challenges exist due to the lack of dedicated programs, hubs or services for young adults with T2D. Thus, there is need for the development of tailored, evidence-based health promotion resources, using an application appropriate to the culture and context, in order to encourage screening uptake among this priority population [19–23].

Best-practice development of health promotion resources targets modifiable behavioural determinants for a clearly specified health behaviour. The UK Medical Research Council (MRC) framework for the design and evaluation of complex interventions recommends use of good quality evidence from a range of sources, strong theoretical underpinnings, causal modelling and a well-designed evaluation [24]. Intervention mapping (IM) is a six-step protocol encompassing MRC elements, which provides an effective and useful framework for this purpose [25]. Key activities are: 1) detailed needs assessment, developing causal logic model of the problem, 2) stating program outcomes and performance objectives, developing logic model of change, 3) utilising theory and evidence-based change methods, designing program to target identified behavioural determinants, 4) producing, pre-testing and refining program with broad stakeholder input, 5) planning for program implementation, and 6) planning for evaluation [26]. Intervention mapping has been widely used by intervention planners to guide the development of effective health promotion materials in a variety of contexts and populations [27–31] and has been shown to be effective both in identifying determinants and increasing uptake for a range of disease prevention interventions [32]. Utilising IM, the aim of the current study was to identify determinants of screening behaviour for young adults with T2D, and develop an engaging psycho-educational resource to target these factors, designed to promote screening uptake.

## Method and results

In this section, IM steps 1–4 are presented in detail, followed by summaries of Steps 5 and 6. Method and results are reported separately for each step, including illustrative examples of key IM activities (with full detail provided in Additional files). Table 1 provides an overview of each IM step as it was applied to this project.

**Table 1:** Overview of IM steps and activities applied to the current leaflet development program

IM steps	IM activities
Step 1: Logic model of the problem	• Establish and work with a planning group
	• Conduct mixed-methods needs assessment
	• Create logic model of the problem
	• Describe context of the intervention and state program goals
Step 2: Program outcomes and objectives; logic model of change	• State expected behavioural outcomes and Performance Objectives (PO)
	• Create logic model of change
	• Create matrix of Change Objectives
Step 3: Program design	• Generate program themes, components, scope and sequence
	• Choose theory and evidence-based change methods
Step 4: Program production	• Draft persuasive message content and leaflet
	• Pre-test, refine and produce leaflet
Step 5: Program implementation	• Identify program implementers, adopters and maintainers
	• Design implementation and liaise with program implementers
Step 6: Program evaluation	• Write effect and process evaluation questions
	• Develop measures for assessment
	• Specify and complete evaluation plan

### Logic model of the problem

#### Establish and work with a planning group

A six-person multidisciplinary planning team was convened comprising representatives from The Australian Centre for Behavioural Research in Diabetes (AJL, JLB, JS); Centre for Eye Research Australia (GR); Diabetes Victoria (CH); and Vision 2020 Australia (DT). Combined, the planning team provided expertise in psychosocial and clinical aspects of diabetes and vision loss, health promotion, behaviour change research methodologies and intervention development. Monthly meetings (chaired by JS) were held throughout the project, with additional meetings held as-needed, and quarterly progress reports provided to the funding body. Throughout the study, the planning team consulted a practicing health psychologist (CA) with expertise in IM and a track record of developing and analysing evidence-based health promotion leaflets [33–36]. Additional expert input was provided by representatives from key stakeholder organisations, such as the National Diabetes Services Scheme (NDSS, an initiative of the Australian Government, which provides free or subsidised self-management supplies and services to registrants), Optometry Australia and key units within Diabetes Victoria.

Patient and public involvement (PPI) is essential for the development of high-quality health behaviour change interventions [37] and is recommended specifically as a strategy for engaging groups at high risk of underutilsation of eye healthcare services [38]. In this study, five young adults with T2D were involved, providing feedback on all study documentation, piloting the quantitative survey and providing detailed review of the eye health leaflet.

#### Conduct mixed methods needs assessment

Our study of the literature (summarised in Additional file 1) revealed that, while there was a paucity of research in this specific area, sub-optimal diabetes self-management (in general) among young people is likely driven by low socioeconomic status [39], low general and health literacy [39], low engagement with diabetes self-management education [20, 39–41], cultural diversity of the priority population [42], optimistic bias and low risk perception [43], life-stage demands [44], high rates of diabetes-related distress [40] and complex healthcare needs [45].

In our empirical needs assessment studies, we sought to determine the relevance of these factors to DR screening specifically, and to identify any additional factors that may facilitate or impede this target behaviour. As other researchers have found it challenging to recruit young adults with T2D to research studies [46, 47], several steps were taken to boost recruitment in the mixed-methods needs assessment. These included: giving priority to ease of participant access; distribution of engaging, cohort-appropriate recruitment invitations with an NDSS and Diabetes Australia branded cover letter introducing the study; reminder invitation after four weeks; age-appropriate incentives (e.g. entry to a technology-based prize draw), and extension of recruitment periods until participant registration visibly flagged.

##### In-depth qualitative interviews

**Qualitative interview procedure**

Detailed description of the study methods and findings, including the participants, procedure, interview guide and analysis, are published elsewhere [44], see Additional file 2 for interview guide. In brief, we conducted in-depth semi-structured interviews to explore factors affecting screening behaviour for young adults with T2D, with an emphasis on those that were individual-level and modifiable. The study was advertised widely online and in community settings, and recruitment invitations were mailed to eligible members of a leading state diabetes consumer advocacy organisation. All interviews were conducted via telephone by an experienced interviewer (AJL), audio-recorded and transcribed verbatim. All transcripts were checked for accuracy and imported into NVivo10 (QSR International Pty Ltd., Doncaster, VIC., Australia, 2012). Transcripts were subjected to content analysis (by AJL), with each participant utterance coded for behavioural determinants (using an a priori coding framework informed by the literature [48]), and again as either ‘facilitator’ or ‘barrier’ dependent upon the context. Twenty percent of transcripts were double-coded (by JLB), with high inter-rater reliability of 99%. Screening determinants were rank-ordered by frequency of coding (higher frequency of utterances interpreted to indicate higher salience).

**Qualitative interview findings**

In brief, ten young adults with T2D (50% women, aged 29–37 years) were interviewed (average length: 55 min, range: 31–106 min). Fifty percent had not attended retinal screening previously. Although young adults with T2D knew of a link between diabetes and vision loss, they did not have a comprehensive understanding of DR or screening (e.g. symptoms, risk factors, screening guidelines, distinction between screening and standard vision checks). Participants reported distress related to having a condition stereotypically associated with older people, and many did not know others of similar age with T2D. Participants indicated that absence of social influence (e.g. prompting from significant others, social comparison with others), and low DR risk perception, combined with life-stage barriers (e.g. lack of time and finances), negatively impacted screening uptake. Concerned about negative judgment by others, and fearing a DR diagnosis, participants reported that they did not always disclose their diabetes diagnosis or proactively seek healthcare or social support, thus losing crucial pathways to timely screening uptake. Irrespective of their screening history, young adults with T2D identified a range of screening barriers, suggesting that a cumulation of factors may impact uptake, thus highlighting the need to acknowledge and address a broad range of barriers in a tailored intervention.

Screening facilitators were often conceptualised by participants as the opposite of the barriers (e.g. improved, as opposed to inadequate, knowledge or access to social support). However, the study also highlighted other screening facilitators: participants compared themselves with others experiencing diabetes-related vision loss, and were thus influenced to engage in screening due to concerns about the impact that vision loss would have on their lives, including anticipated regret at the potential impact on their spouses and/or children. For those who previously attended screening, feelings of relief and reassurance facilitated repeat screening behaviour, with participants expressing intent to sustain the behaviour and expectation of a positive outcome (i.e. no DR diagnosis).

##### National online survey

**Online survey procedure**

*Survey development*

Using the Information-Motivation-Behavioural skills (IMB) model [49] as a foundation, the planning team developed a survey designed to identify modifiable behavioural determinants for screening. The IMB model posits that although information is a key element in changing behaviour, increasing knowledge and awareness of a behaviour is not sufficient in itself, and requires the integration of motivational and skills elements to ensure behaviour change. Use of the IMB model in behaviour change research requires identification of deficits in each of the three key areas, to be addressed in a subsequent intervention. The IMB model has been effective both as a framework for intervention design [50] and as a predictive model for health-related screening behaviours, such as breast self-examination [51].

Increasingly used with chronic conditions, the IMB model has been validated recently in a model of diabetes self-care behaviours [52] and medication adherence [53]. Survey items were based on IMB-based questionnaires previously validated for diabetes self-management [52, 53], the widely-used Theory of Planned Behaviour [54], and cognitive constructs shown to be relevant to young adults with T2D (e.g. optimism/fatalism, social support, risk perception, anticipated regret, self-efficacy) [43, 55].

In brief, the survey comprised 54 items assessing information/knowledge, motivation and behavioural skills (see Additional file 3 for individual items). **Information:** 16 items assessed knowledge of the link between diabetes and vision loss, diabetic retinopathy and retinal screening. Responses scored dichotomously (incorrect / correct). **Motivation:** 21 items collectively assessed three attitudinal constructs (attitudes toward screening for DR, perception of personal risk, and anticipated regret); three items assessed normative beliefs and three items assessed intention. **Behavioural skills:** 11 items collectively assessed two behavioural skills constructs (perceived control over screening and overcoming barriers).

Unless otherwise noted, each item was rated on a 7-point Likert scale (*“strongly disagree”* to *“strongly agree”*). Individual items were aggregated to provide a composite score for each construct, with good internal consistency (see Additional file 3). For each, higher scores indicated greater endorsement of the construct measured (e.g. stronger intentions, more positive attitudes). In addition, we collected socio-demographic data to describe the sample at baseline. The survey was piloted with young adult PPI members and representatives from selected stakeholder organisations, who also commented on readability, format, accessibility and content; no substantive changes were required.

*Data collection and participants*

The survey was conducted nationwide and hosted via a secure online survey platform, Qualtrics™ (Provo, UT, 2014–2015). In Australia, the majority of people with a confirmed diagnosis of diabetes are registered by their health professional with the NDSS [56]. All young adults with T2D who had been registered on the NDSS in the previous three years (registration date was used as a proxy for diagnosis date), and who had consented to be contacted for research (*N* = 5354) were invited to participate. Exclusion criteria included non-English speaking; those aged 40+ years, and diagnosis of another type of diabetes. Study invitations were managed by the NDSS in order to preserve registrant confidentiality, but purposive sampling of those who had not previously screened for DR was not possible, due to lack of available data on retinal screening status of NDSS registrants. Recruitment to the online survey continued for seven weeks.

*Statistical analyses*

Statistical analyses were conducted using SPSS version 22 (SPSS Inc., Chicago IL, USA). Univariate analyses (chi-square and independent measures t-tests, two-sided) were conducted to explore between-group (previous retinal screen: yes/no) differences on demographic variables and modifiable behavioural determinants at the item level (to inform specific intervention message content). Given the large number of analyses, a conservative *p* < 0.01 was considered statistically significant.

**Online survey findings**

Overall, 129 participants (2% of eligible population) completed the full survey, and their sociodemographic characteristics are presented in Table 2. Sixty percent were women, average age 34 ± 5 years (range: 19–39 years), and 74% had previously screened for DR. No significant differences in sociodemographic characteristics were found between screening groups.

**Table 2:** Sociodemographic characteristics by screening behaviour (*N* = 129)

Sociodemographic characteristics	Retinal screen		*p*-value
	No (*n* = 33)	Yes (*n* = 96)	
Age, years	34.39 (33, 37)	34.04 (32, 37)	.697
Duration, years	1.00 (1.84)	1.69 (1.97)	.081
Gender: women	15 (45)	62 (65)	.084
Primary diabetes management			
Lifestyle only	5 (15)	21 (22)	
Medication (not insulin)	23 (70)	64 (67)	.652
Insulin	5 (15)	11 (11)	
Country of birth: Australian born	18 (55)	66 (69)	.206
Main language spoken at home: English	27 (82)	81 (84)	.944
Employment status: employed	20 (61)	57 (59)	1.000
Socioeconomic status^a^	984.55 (83.52)	991.48 (57.11)	.660
Family history of T2D^b^	22 (67)	72 (75)	.483
≥1 comorbid health condition^b^	25 (76)	75 (79)	.891
Depression (PHQ-2)^c^	2.94 (2.48)	2.12 (2.07)	.102

Data are number (%), mean (SD), or median (IQR); *p-value* is Pearson’s chi-square or independent t-tests (two-sided); statistical significance *p* < 0.05

^a^Index of Relative Socio-economic Advantage and Disadvantage: lower score indicates relatively greater disadvantage, range 300–1250

^b^Missing data (average 6%, range 2–11%)

^c^PHQ-2 range 0–6: ≥3 indicating likely depression

*Behavioural determinants of screening*

Selected findings for information (knowledge), motivation and behavioural skills items are detailed in Table 3 (full detail and construct-level findings provided in Additional file 3).

**Table 3:** Selected behavioural determinant items by retinal screen (*N* = 129)*

Modifiable behavioural determinants	Retinal screen		*p-*value
	No (*n* = 33)	Yes (*n* = 96)	
INFORMATION (KNOWLEDGE) ITEMS			
Diabetes can lead to vision loss	30 (91)	93 (97)	.174
All people with diabetes are at risk of DR	26 (79)	89 (93)	.004
Recommended target HbA1c^a^	17 (53)	81 (87)	<.001
Initiate eye examinations ‘at diabetes diagnosis’	5 (15)	42 (45)	.004
Screen ‘at least every 2 years’ if no DR present	0 (0)	18 (19)	.003
MOTIVATION ITEMS^b^ An eye health check for DR would be...^c^			
...(not) ‘unpleasant’	3.71 (0.94)	3.86 (1.07)	.500
...reassuring	3.94 (0.96)	4.63 (0.61)	<.001
...important	4.06 (1.06)	4.89 (0.35)	<.001
...empowering	3.10 (1.19)	3.73 (0.97)	.004
...comfortable	3.26 (1.15)	3.68 (1.10)	.073
I believe I will develop DR due to my diabetes^d^	4.03 (1.45)	4.14 (1.62)	.734
Expect to be diagnosed with DR at next eye check^d^	2.97 (1.47)	2.43 (1.66)	.114
Can reduce risk of vision problems...^d^	2.32 (1.44)	1.43 (0.79)	.002
If I did NOT have an eye health check for DR, I would feel...^d^			
...concerned	5.03 (1.70)	5.88 (1.40)	.007
...fearful	4.48 (1.79)	5.13 (1.70)	.073
...worried	4.65 (1.80)	5.53 (1.47)	.007
My family/close friends would approve...^d^	5.94 (1.69)	6.82 (0.80)	.008
I plan to attend an eye health check...^d^	4.26 (2.32)	6.76 (0.77)	<.001
I intend to have an eye health check...^d^	4.42 (2.32)	6.74 (0.77)	<.001
BEHAVIOURAL SKILLS ITEMS^b,e^ How confident are you that you...			
...know steps to reduce the risk of developing DR	2.39 (1.17)	3.06 (1.29)	.012
...will remember to have an eye health check…	2.68 (1.35)	4.36 (0.90)	<.001
...can talk to your doctor about your eye health	3.39 (1.28)	4.17 (1.03)	.001
...can find the time to attend an eye health check…	2.74 (1.37)	4.55 (0.75)	<.001
...can afford to pay for the eye health check…	2.68 (1.49)	3.52 (1.48)	.008

*DR* diabetic retinopathy. Data are number (%) of participants who answered each item correctly (Knowledge items); mean (SD) Motivation and Behavioural skills items. *p-value* is Pearson’s Chi-Square (or Fisher’s exact test if expected cell count< 5), or Independent-samples t-test (two-sided); statistical significance *p* < 0.01

*Full detail and construct-level findings provided in Additional file 3

^a^Glycated haemoglobin (measure of average blood glucose over the past 8–12 weeks, and indicator of DR risk)

^b^Valid *n*: 121 (motivation items), 120 (behavioural skills items)

Item response range:

^c^1 (Strongly disagree) to 5 (Strongly agree)

^d^1 (Strongly disagree) to 7 (Strongly agree)

^e^1 (Not at all confident) to 5 (Extremely confident)

Almost all participants (irrespective of previous screening behaviour) knew of a link between diabetes and vision loss. However, compared to their non-screening counterparts, those who had previously screened knew that all people with diabetes were at risk of DR, the clinically-recommended HbA1c (average blood glucose) target for DR prevention, when to initiate screening and recommended screening intervals.

Overall, participants who had screened indicated more positive attitudes towards the behaviour (e.g. empowering, reassuring and important) than those who had not screened. No differences were observed between groups on how pleasant or comfortable the eye check was perceived to be, although scores were lower for all participants compared to other attitude items. Perception of personal risk of vision problems and DR were moderate for all participants with low expectations of a DR diagnosis in the short term. Although all participants believed they could not reduce their risk of vision problems, those who had screened held this belief more strongly. All participants reported negative emotions when thinking about not screening, including fear, which was high for both groups. Compared to their non-screening counterparts, those who had previously screened reported greater concern and worry at the prospect of not screening. Participants who had previously screened were significantly more likely to agree that significant others (i.e. family/friends, healthcare team) would approve of screening. Intention to screen was high among all participants but significantly higher for those who had previously screened compared to those who had not.

Those who had screened previously reported significantly greater confidence on all aspects of behavioural control over screening (e.g. how to make an appointment for screening, ability to screen regularly, remember and attend an appointment). No differences were observed between groups on confidence in knowing the steps that can be taken to reduce the risk of DR, although scores were lower for all participants compared to other behavioural control items. Those who had screened also reported significantly higher confidence in overcoming common screening barriers (including time and cost, and discussing diabetes and DR with healthcare professionals).

##### Summary of key learnings from needs assessment

Key learnings from the literature review, qualitative interviews and quantitative survey are summarised in Table 4. The survey identified that compared to their non-screening counterparts, those who had previously attended screening reported: significantly higher knowledge of both DR and retinal screening; more positive attitudes towards screening; stronger agreement that significant others would approve of the behaviour; higher intention to screen; greater perceived behavioural control (i.e. confidence that they could arrange and attend screening when due), and greater confidence in addressing common screening barriers.

**Table 4:** Key lessons learned from needs assessment

Compared to their older adult counterparts, young adults with T2D have different psychosocial and information needs. There is a lack of behavioural interventions focused on encouraging screening uptake among young adults with T2D, indicating that development of a tailored intervention is warranted. Perceived barriers to and facilitators of screening (which are modifiable and within the scope of the current intervention) include: • Knowledge: diabetic retinopathy (awareness of asymptomatic nature of DR, high personal DR risk, modifiable risk factors), screening (consequences of not screening, role of screening in early detection of DR and subsequent benefit of timely treatment, distinction between standard eye check and retinal screen) • Attitudes: low perception of personal risk, recognition of the benefit of screening • Normative beliefs: awareness of screening approval by significant others, and screening approval and behaviour of similar others • Intention: low prioritisation of target behaviour • Behavioural skills: self-efficacy in overcoming common screening barriers to ensure screening attendance (e.g. lack of time or resources), engagement with healthcare (sharing diabetes diagnosis, participation in diabetes self-management behaviours)	

The findings suggest that messages highlighting the prevalence of DR and link between DR and diabetes duration are warranted to prompt reassessment of personal risk. Information on modifiable DR risk factors (blood glucose, blood pressure and cholesterol), asymptomatic nature of the condition and screening guidelines are needed to encourage individuals to both reduce DR risk and initiate screening.

Messages designed to highlight the health and material consequences of screening, including likely positive emotional consequences, are warranted in order to promote positive screening attitudes. Findings suggesting that all participants perceived screening as potentially ‘unpleasant’, ‘uncomfortable’ and disruptive to normal activities are realistic considering that many people experienced discomfort and delay from pupil dilation (mydriasis) drops. Consequently, positive messages should be balanced by acknowledgement of the potential for negative consequences related to mydriasis in order to maintain credibility.

Although moderately high levels of distress in the priority population mean that it is important to avoid direct ‘fear appeal’ messages, low anticipated regret scores for those who had not screened reinforce the need for messages which emphasise personal susceptibility and describe the likely consequences of not screening. Similarly, responses to risk perception items point to a possible unrealistic level of optimism, highlighting the need to emphasise personal susceptibility while providing information-based content on steps that can be taken to reduce DR risk. As with many other preventive behaviours, awareness of the potential effectiveness of screening followed by subsequent protective action did not necessarily result in intention formation or prioritisation of preventive intentions. Cognitive dissonance induction techniques have been found to have generally positive effects on changing attitudes, motivations and health-related behaviour patterns [57]. Consequently, we selected dissonance reduction as a technique that could promote screening motivation.

Responses to normative behaviour items suggest that messages that provide information about significant others’ approval are warranted. The findings suggest that inclusion of procedural information and messages to promote confidence in knowing steps that can be taken to reduce DR risk including how to book and remember a retinal screen, as well as overcoming common barriers are warranted. Emphasis is required to minimise misconceptions about some barriers (e.g. inclusion of messages which accurately describe the cost and time taken for the procedure).

#### Logic model of the problem

**Fig. 1:**
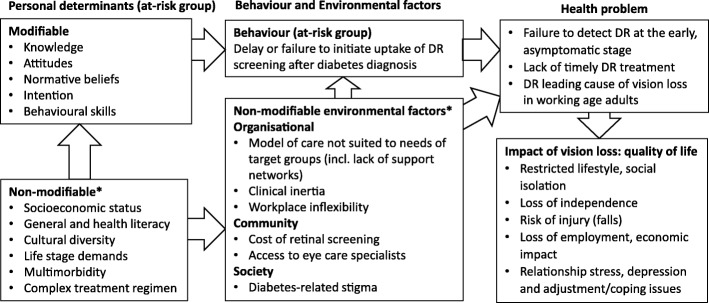
Logic model of the problem DR: diabetic retinopathy; *Identified in the needs assessment but cannot be modified by the current intervention

Giving consideration to both the qualitative and quantitative needs assessments, we synthesised our findings into a logic model for DR screening. The aim of the logic model was to identify the pathways of problem causation moving from determinants, to low screening rates and consequent impact on health and quality of life (Fig. 1).

#### Context of the intervention and program goals

The intervention was to be evaluated and implemented in a real-world setting where intervention format and delivery medium were dictated by broader policy-level initiatives and a fixed delivery timeline. The intervention was funded by Vision 2020 Australia and grounded within a suite of Vision Initiative projects collectively designed to achieve the aims of the Commonwealth government ‘National Framework for Action to Promote Eye Health and Prevent Avoidable Blindness and Vision Loss’ [58]. Vision Initiative policy required that the resource be targeted at the individual-level and delivered directly to eligible young adults with T2D (NDSS registrants). As such, it was determined by the planning team members who were involved in conception of the study, that the most efficient, cost-effective way to meet these criteria was for the intervention to take the form of a leaflet, to be posted to eligible NDSS registrants. Furthermore, this enabled the leaflet to be included in future ‘NDSS starter packs’ for new registrants, and to be made available online. This decision was supported by previous research, which showed that printed materials are acceptable to young adults with T2D, who give preference to consistent, centralised information over format, and who specifically state that the NDSS ‘starter pack’ is a useful resource.

With 86% of Australians with T2D registered, the NDSS is considered the “best available source to monitor type 2 diabetes in children and young people in Australia” [p.36, 56]. However, the NDSS database primarily records registrant postal addresses, which necessitated the use of a print-based intervention tool that could be posted to registrants.

Overall, the purpose of the intervention was to promote uptake of screening among young adults with T2D. Accounting for real-world logistical considerations, the program goal was to develop a leaflet intervention that: could be delivered by post, was tailored to young adults with T2D, and included persuasive messaging targeting behavioural determinants of the target behaviour.

### Program outcomes and objectives; logic model of change

#### Expected behavioural outcomes and performance objectives

The multidisciplinary planning team defined a single, measurable primary outcome when planning for the subsequent evaluation (uptake of screening for those young adults with T2D who had not previously screened for DR), and multiple secondary outcomes (i.e. change in nominated modifiable behavioural screening determinants).

Working from the designated program outcomes, and informed by the findings of the needs assessment, the planning team established the foundation for the intervention by defining four Performance Objectives (PO, Table 5). We increased the specificity of each Performance Objective by defining sub-objectives, each identifying a behaviour or cognitive process that would promote screening uptake.

**Table 5:** Performance Objectives (e.g. PO.1) and sub-objectives (e.g. PO.1.1, PO.1.2, etc.)

Young adults with type 2 diabetes will…	
PO.1.… demonstrate a clear understanding of diabetic retinopathy (DR)	
PO.1.1 Modifiable and non-modifiable DR risk factors	
PO.1.2 Clinical targets for reducing risk of DR	
PO.1.3 Symptoms of DR	
PO.1.4 Role of DR in vision loss	
PO.1.5 Prevalence of DR	
PO.2…demonstrate clear understanding of retinal screening	
PO.2.1 Role in detecting DR and reducing vision loss	
PO.2.2 Screening procedure and experience	
PO.2.3 Booking and examination procedure	
PO.3… be motivated to engage in retinal screening	
PO.3.1 Prioritise retinal screening	
PO.3.2 Understand personal risk of DR	
PO.3.3 Identify personal barriers to retinal screening	
PO.3.4 Perceive personal responsibility to engage in screening	
PO.4…proactively engage with the healthcare system and their healthcare team	
PO.4.1 Discuss diabetes and eye health with healthcare professionals	
PO.4.2 Understand treatment benefits and options	
PO.4.3 Seek more information about diabetes and eye health	

#### Create logic model of change

**Fig. 2:**
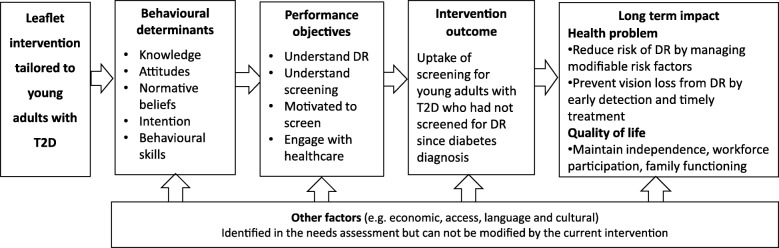
Logic model of change T2D: type 2 diabetes, DR: diabetic retinopathy

We developed a logic model of change (Fig. 2) to depict the hypothetical causal pathway from the intervention to program outcomes, and anticipated health and quality of life improvements. Commencing with the intervention, we outlined the five modifiable behavioural determinants (from Fig. 1) and four Performance Objectives (Table 5), which were expected to change the measurable behavioural outcome. The planning team also acknowledged external factors that may affect screening behaviour (i.e. factors that cannot be changed through an individual-level intervention) in the logic model, even though these were beyond the scope of our intervention.

#### Create matrix of change objectives

Once the health behaviours, Performance Objectives and determinants were defined, Change Objectives were developed. Change Objectives are integral to intervention content because they represent the behaviour or cognition being targeted. A sub-group of the planning team generated Change Objectives by creating a matrix, with modifiable behavioural determinants (in columns) and sub-objectives (in rows).

Table 6 presents a matrix of Change Objectives for Performance Objective 3 (Young adults with type 2 diabetes will be motivated to engage in retinal screening). To illustrate, two Change Objectives were generated for sub-objective 3.2 (Understand personal risk of DR), at the intersection with two determinants (knowledge and attitudes). The first (K.3.2) sought to improve knowledge that risk of DR increases over time, and the second (A.3.2) sought to change attitudes regarding personal risk and susceptibility to DR. See Additional file 4 for a complete matrix of Change Objectives.

**Table 6:** Illustrative matrix of Change Objectives for Performance Objective 3 (PO.3) - Young adults with type 2 diabetes will be motivated to engage in retinal screening^a^

Sub-objectives^b^	Modifiable behavioural determinants				
	Knowledge	Attitudes	Normative beliefs	Intention	Behavioural skills
PO.3.1 Prioritise retinal screening			NB.3.1 Recognise that similar others have overcome screening barriers	I.3.1 Form an intention to prioritise retinal screening	
PO.3.2 Understand personal risk of DR	K.3.2 Know that DR risk increases over time	A.3.2 Perceive high personal risk and susceptibility to DR			
PO.3.3 Identify personal barriers to retinal screening		A.3.3 Believe that attending screening will relieve fear and guilt and be a positive experience	NB.3.3 See that similar others face screening barriers (e.g. cost, fear of adverse effects)		BS.3.3 Be confident in one’s ability to identify and overcome common screening barriers
PO.3.4 Perceive personal responsibility to engage in screening	K.3.4 Know that they can take steps to protect eye health	A.3.4 Adopt personal responsibility for retinal screening	NB.3.4 Believe that similar others take responsibility for their own eye health		BS.3.4 Be confident they have the tools to act on personal responsibility

Determinants: K=Knowledge, A = Attitudes, NB=Normative Beliefs, I=Intention, BS=Behavioural Skills

*PO* performance objective, *DR* diabetic retinopathy

^a^Full matrix of Change Objectives for every Performance Objective is provided in Additional file 4

^b^See Table 5 for full list of Performance Objectives and sub-objectives

### Intervention design

#### Intervention themes, components, scope and sequence

Ensuring that all components of the intervention reflected the needs and preferences of young adults with T2D was a crucial consideration for the planning team. The health behaviour change and health communication literature provided ample foundation on best practice presentation of message content [20, 25, 40, 59–64]. Informed by this evidence and the findings of the needs assessment, we developed seven guiding principles for leaflet intervention design (Table 7). Consultation with young adult PPI members and experts from key stakeholder groups confirmed that these were appropriate guiding principles from their perspective.

**Table 7:** Guiding principles for retinal screening leaflet intervention design

Readability and comprehension: content to be written to acceptable (health) literacy standards, with minimal technical or medical terminology [59, 60].	
Scope: the scope of intervention messages to be restricted to targeting individual-level, modifiable behavioural determinants.	
Framing: despite long term benefit, retinal screening can be considered a high-risk behaviour due to the potential for immediate DR diagnosis [61]. Loss-framed messages are effective in promoting engagement with high-risk behaviours and will be used in this leaflet [25]. The majority of headings to be framed as questions to engage the reader while minimising any potentially defensive reaction [62].	
Sequence: content to follow the logical order of reading. In order to balance loss-framed messages against the high levels of diabetes-related distress and anxiety experienced by young adults with T2D [20, 40], potentially threatening content to be immediately followed by an empowering or reassuring statement.	
Use of quotes: in recognition of the subtle aspects of social influence, where an individual’s’ beliefs are influenced by those accepted and encouraged by the majority [63], quotes from similar others to be used to reinforce key persuasive messages. All quotes to be sourced verbatim from interview study with descriptors (age and diabetes duration) included to reinforce group membership.	
Credibility: quote descriptors within the leaflet to reflect demographic characteristics of the priority population to prompt identification with a credible source. Similarly, logos of leading diabetes and eye health organisations that had contributed to the content to be included to enhance credibility of information. Important yet necessary negative information (e.g. discomfort associated with mydriasis, time required to recover clear vision) to be included to provide balance.	
Graphics and imagery: to reflect the demographic characteristics of the priority population (e.g. young adults from a range of ethnicities, with and without children). National interpreter symbol to indicate availability of language assistance services to those with limited English proficiency [64].	

#### Choose theory and evidence-based behaviour change methods

Having established guiding principles, the planning team selected types of theory-based psychological change techniques (or change strategies) [65, 66] grouped into six broad change mechanisms designed to ‘boost motivation and prompt action’ ([65], p.104). The constituent techniques (or practical methods) included in the leaflet focused on: i) changing beliefs about the benefits of screening (e.g. providing general information on behaviour-health links, describing likely consequences of behaviour); ii) changing risk perception (e.g. emphasising personal susceptibility to negative consequences, prompting recipients to assess own risk); iii) changing attitudes associated with screening uptake (e.g. describing likely emotional or affective consequences, potentially inducing cognitive dissonance among those not intending to act in the face of negative consequences); iv) changing (normative) beliefs about others’ behaviour (e.g. emphasising significant others’ approval of screening behaviour, providing information about others’ screening behaviour); v) fostering a positive screening identity (e.g. providing a positive group identity for those engaging in screening); and vi) enhancing self-efficacy (e.g. using persuasive argument to bolster self-efficacy, providing instruction, prompting barrier identification and planning in relation to anticipated barriers, prompting goal setting).

### Intervention development

#### Draft persuasive message content and leaflet

##### Develop message content

Working from the matrix of Change Objectives, the guiding principles, and theory and evidence-based intervention strategies noted above, a pool of more than 60 persuasive messages was developed. From this pool, specific change techniques or practical methods were selected to encourage screening. For example, to achieve Change Objective A.3.4 (View retinal screening as a personal responsibility), four potential leaflet heading messages were developed: ‘Eyes: they’re important any way you look at it’, ‘The only way to know is to go…’ (verbatim quote), ‘Who is looking after your eyes?’, and ‘Looking at the facts’. All messages were reviewed by the planning team, and a sub-set selected based on the perceived capacity of the message to achieve program goals, target individual Change Objectives, and satisfy the leaflet guiding principles. Thus, in the above example, the third option (‘Who is looking after your eyes?’) was selected because it was phrased as an engaging question, promoting personal responsibility with potential to reduce defensive reaction while motivating screening.

A selected example, linking leaflet content to Performance Objective 3, is presented in Table 8. Full intervention map detail for all Performance and Change Objectives is provided in Additional file 5.

**Table 8:** Illustrative intervention map linking leaflet content directly back to Performance Objective 3 (PO.3: Young adults with T2D will be motivated to engage in retinal screening)

Sub-objective (i.e. 3.1) and related Change Objectives^a^	Leaflet content (antecedent leaflet text in brackets illustrates context)	Panel number^b^
**PO.3.1**	**Prioritise retinal screening**	
NB.3.1 Recognise that similar others have overcome screening barriers	(Jenny’s story: before and after the eye health check). “I was scared. I was scared of what damage was done…of confronting the fact that my eyesight could be damaged, and of going through the exam and being confronted with what’s there.”	7
I.3.1 Form an intention to prioritise retinal screening	(What can I do to protect myself from DR and prevent vision loss?) 1. Have a diabetes eye health check. (Note: eye health check listed as Step 1, highest priority)	5
**PO.3.2**	**Understand personal risk of DR**	
K.3.2 Know that DR risk increases over time	• The longer you have diabetes the more at risk you are of DR	1
A.3.2 Perceive high personal risk and susceptibility to DR	Image: mother and daughter smiling. Child holding hands over mother’s eyes But I’m still young. Am I at risk of DR?	1
	Yes you are. Anyone with diabetes can develop DR, which is the leading cause of vision loss for people under 60 years.	1
	There are over 34,000 Australians with type 2 diabetes who are under 40 years of age. More than 8500 will already have DR.	1
	• The longer you have diabetes the more at risk you are of DR. (Lucas, aged 34, diagnosed with type 2 diabetes 2 years ago)	1
	“I didn’t know that I was at risk.” (Jane 25 years, diagnosed with type 2 diabetes 3 years ago)	1
	“You might have good vision, you might think that your eyes are absolutely brilliant and there’s no issue. But in the back of your eye, there could be a problem with those little tiny veins that you don’t realise.”	4
**PO.3.3**	**Identify personal barriers to retinal screening**	
A.3.3 Believe that attending screening will relieve fear and guilt and be a positive experience	(Jenny’s story: before and after the eye health check). “It was actually quite fun; I don’t know why I put it off. I was really scared going in there, but definitely not now – I’m not fazed by it at all.”	7
NB.3.3 See that similar others face screening barriers (e.g. cost, fear of adverse effects)	(Jenny’s story: before and after the eye health check). “The eye drops were a bit uncomfortable and there was a small cost – but I think it’s a wise spend considering what you’re preventing.”	7
BS.3.3 Be confident in one’s ability to identify and overcome common screening barriers	(What else do I need to know?) A diabetes eye health check takes about 30 minutes.	6
	(What else do I need to know?) It may be free (bulk-billed) or there may be a small fee.	6
	(What else do I need to know?) Your optometrist may use eye drops which helps them to see the back of your eye. If you do have eye drops, they may be a little uncomfortable. The drops will also leave you sensitive to light, so bring your sunglasses and be prepared to wait a while for your vision to return to normal	6
**PO.3.4**	**Perceive personal responsibility to engage in screening**	
K.3.4 Know that they can take steps to protect eye health	What can I do to protect myself from DR and prevent vision loss?	5
A.3.4 Adopt personal responsibility for retinal screening	“I’m a busy person and my family depend on me.”	1
	Leaflet heading: Who is looking after your eyes?	3
NB.3.4 Believe that similar others take responsibility for their own eye health	Images: mother and daughter, smiling couple selfie, young man of indeterminate cultural origin, Asian female (a.k.a. ‘Jenny’)	1,3,5,8
BS.3.4 Be confident they have the tools to act on personal responsibility	Leaflet sub-heading: Your guide to preventing vision loss from diabetes eye disease	3
	Protect your sight for life	2

Complete intervention map for all Performance and Change Objectives is provided in Additional file 5

Determinants: K = knowledge, A = attitudes, NB = normative behaviour, I = intention, BS = behavioural skills

*PO* Performance Objective (in bold), *DR* diabetic retinopathy

^a^See Table 6 for illustrative matrix of Change Objectives and Additional file 4 for complete matrix

**Fig. 3:**
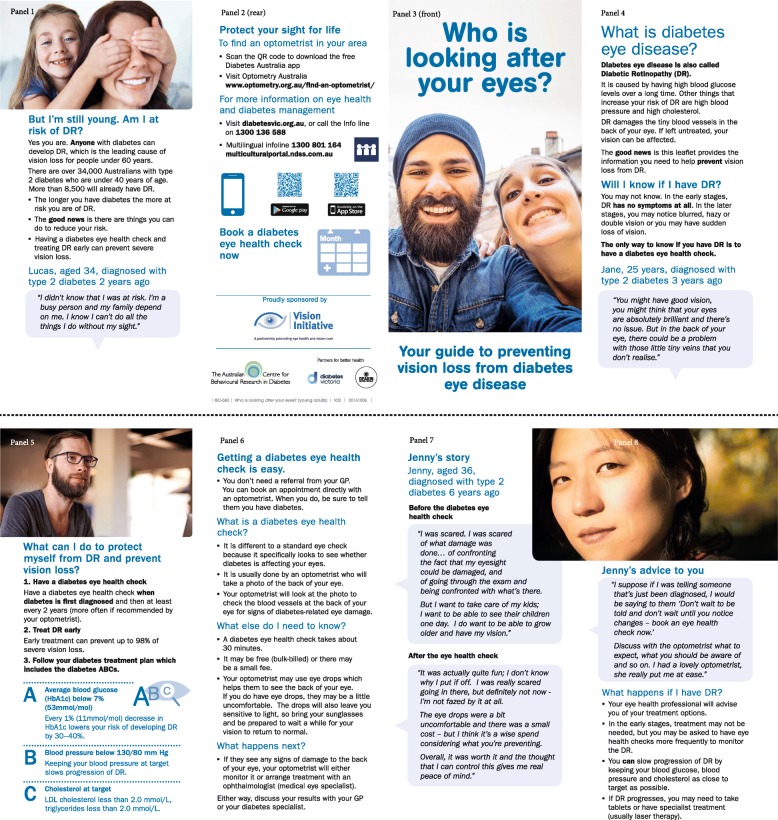
Who is looking after your eyes? tailored leaflet. ©Vision 2020 Australia, 2018. All rights reserved

^b^See Fig. 3 for leaflet panels

##### Assessing readability and suitability

The leaflet was assessed using a combination of an online readability consensus calculator and the Suitability Assessment of Materials (SAM) test, consistent with best practice [67]. The consensus calculator reports synthesised results from seven assessment tools (e.g. Flesch Reading Ease formula, Flesch-Kincaid Grade), to provide two composite scores by grade (range: 4–9) and reading level (range: 0–29 ‘very confusing’ to 90–100 ‘very easy’) [68]. The SAM test uses six evaluation criteria (content, literacy demand, graphics, layout and type, learning stimulation and motivation, cultural appropriateness) to determine overall suitability [69], with scores summed and converted to a percentage score and classified as ‘not suitable’ (0–39%), ‘adequate’ (40–69%), or ‘superior’ (70–100%).

We excluded the front and back panels of the ‘Who is looking after your eyes?’ leaflet from assessment, as they included minimal text. For the remaining panels, the median readability consensus grade was 6; median reading ease level was 75.6 (fairly easy), and SAM test outcome was 75% (superior).

##### Draft intervention materials

The planning team selected an 8-panel leaflet design, with panels opening outward from the centre, which could fit into a standard DL-size envelope. A range of leaflet design options were created in close consultation with a graphic designer who had expertise in producing health promotion materials for people with diabetes. The designs varied in structure, imagery and organisation, but all adhered to the guiding principles and included consistent messaging.

#### Pre-test, refine and produce leaflet

##### Validation and pilot testing

The draft leaflet was reviewed by the planning team and representatives from key stakeholder organisations to confirm that all content was factually accurate and clinically appropriate, and that the resource was likely to meet the project objective. Young adult PPI members participated in a thorough piloting process to determine whether: the images and quotes were culturally relevant and resonated with the reader; participants perceived the leaflet would have met their information needs at the time of their T2D diagnosis; and there were any unintended adverse effects in the messaging, imagery or format. Each young adult PPI member received the draft leaflet by post and, after reviewing it, participated in a telephone interview during which they commented on the leaflet’s suitability, responding to questions based on the SAM criteria [69].

Feedback from stakeholder reviewers was positive, with minimal critique offered. Young adult PPI members gave more considered commentary on what they found useful and what could be improved (Table 9). Where appropriate, the leaflet was revised to improve content, imagery, readability and cultural acceptability. Once finalised, leaflet printing was managed by Diabetes Victoria (the state agent of the NDSS).

**Table 9:** Suitability Assessment Materials (SAM) evaluation criteria, young adults’ feedback and changes made to leaflet

Sample pilot questions	Young adults’ feedback	Changes to leaflet
Content: Do you think that this leaflet achieves the purpose of the project? • Did you learn anything new?	“Key information came through really clearly. I didn’t know that early DR doesn’t have any symptoms…the doctors tend to focus on blood glucose, so I knew the 7% (HbA1c) but I didn’t know what the cholesterol target and normal blood pressures were.” ID32 “This leaflet improved my intentions. (DR) is not something you would think could happen to young people.” ID32 “‘Protect your sight for life’ is a powerful statement.” ID36	Make ‘Protect your sight for life’ a stand-alone statement and place at top of panel 2, which signposts location of more information
Literacy demand: Was the length of the leaflet acceptable to you? • How about the number of words? • How easy was it to read and understand the information in the leaflet? • Are the words used simple, clear and informal? • Were medical terms defined adequately?	“It only took about 5 minutes to read.” ID33 “Language is pretty relaxed which is good for young people.” ID40 “The only thing that caught me was ‘DR’. Did you mean ‘doctor’ or ‘diabetic retinopathy’? I think you should bold it when it is first defined.” ID32	Discuss whether to include ‘DR’ in leaflet. By consensus, a decision was made to include it, but to bold initial definition of diabetic retinopathy and DR acronym at top of panel 4.
Graphics: What do you think of the front panel image? • Are the other images and graphics ‘friendly’ and relevant?	“Very professional. Looks like it’s targeted at my demographic.” ID36 “Maybe bold ‘When diabetes is first diagnosed’ so that you hammer home that it’s never too early to have an eye check.” ID32 “I’ve never really looked at a graph in a pamphlet. It might appeal to some people, but I don’t know…” ID40	Bold text ‘When diabetes is first diagnosed’ in panel 5. Remove graph, which depicted rate of DR progression over time.
Layout and typography: What do you think of the sequence of information? • Is the text type size and font easy to read, or could it be easier? • Is the information in the leaflet well-spaced, or does it appear cluttered or confusing?	“Main headings need to be in a larger sized font and bold, and sub-headings in smaller font. Keep the blue colouring.” ID39 “Is there a way that you can make more white space? The different colours are more attractive.” ID33	
Learning stimulation, motivation: Thinking back to when you first were told that you had diabetes or when you learnt that diabetes could affect the eyes – would the leaflet have met your information needs at that time? • Do you feel that the leaflet is friendly or formal? • Do you feel like you want to read it now or later? Why?	“Jenny’s story is a good thing to have in there. Including name, age and diabetes duration makes the quotes more meaningful.” ID39 “Wow, that looks awesome…I didn’t expect to see two smiling faces on the front because most diabetes things are all doom and gloom, they’re so terrifying and then you don’t want to read them. Whereas, I read this and thought, this was a reminder for me to book in for my eye check.”ID40 “I loved the ‘What happens if I had DR’ section. I kept putting off an eye check because I was scared of what would happen. Can you add more about what the treatment is?” ID32	Revisit Performance Objectives to include understanding the treatment options (PO.4.2), populating this through the matrix of Change Objectives and into the leaflet content. Add more treatment detail to panel 8.
Cultural appropriateness: Do the quotes represent key emotions or experiences that you have felt about eye examinations? Was the language used throughout the leaflet familiar and culturally appropriate to you? • Were there any sections that you found confusing or were unsure about?	“I love the pictures; they speak to different cultural backgrounds.” ID32 “English is not my first language, but I didn’t have any problem reading the leaflet.” ID33	Retain multicultural imagery.

### Intervention implementation

Planning for program adoption and implementation started at study commencement and was heavily influenced by contractual obligations with the funder. These included a one-off statewide distribution of the leaflet to all eligible NDSS registrants, timed to coincide with Vision 2020 Australia public awareness campaign.

To protect registrants’ privacy, the NDSS distributed the final leaflet (presented in Fig. 3) directly to members of the priority population, on behalf of the planning team. Plans are underway for a revision of the NDSS ‘starter pack’ to include the eye health leaflet for young adults with T2D, ensuring long-term sustainability of the intervention. Further, to enhance reach, an electronic copy of the leaflet was made freely available via Diabetes Victoria and Vision 2020 Australia [70, 71] and promoted to healthcare professionals and members of the priority population.

### Planning for intervention evaluation

Similarly, evaluation planning started at study commencement. The planning group determined that the best method of evaluation of the leaflet intervention was a two-arm, wait-list randomised controlled trial with screening uptake as the primary outcome and change in modifiable behavioural determinant constructs as secondary outcomes. The trial, registered with the Australian and New Zealand Clinical Trials Registry (ACTRN12614001110673), is now complete, and a manuscript is in preparation.

## Discussion

Uptake of retinal screening from diabetes diagnosis is crucial for the early identification of DR. In this study, we undertook the systematic development of an evidence-based health behaviour change intervention tailored to the needs of a priority population, young adults with T2D, who are at risk of low retinal screening uptake and vision loss from DR.

To date, lack of information on the determinants of retinal screening behaviour among young adults with T2D, and on the elements of individual-level DR screening interventions [72], has hampered development of effective, targeted intervention strategies for this priority population. Further, previous print-based retinal screening interventions have been limited in focus, aiming primarily to increase knowledge and awareness of DR, and of retinal screening, and neglecting to target other behavioural determinants, such as social norms and intentions [72, 73], despite the acknowledged role of psychosocial factors in health behaviour [74].

The needs assessment described here is the first large-scale, mixed-method exploration of modifiable behavioural factors impacting retinal screening behaviour among young adults with T2D. The findings highlighted that many of the clinical and psychosocial barriers to diabetes self-management faced by young adults with T2D more broadly [18, 20, 40, 41, 75–78], also apply to retinal screening. Importantly, when compared to older adults with T2D, young adults with T2D face both an accumulation of barriers to retinal screening, and a number of uniquely salient barriers and facilitators [44], warranting tailored intervention.

Combined with consensus-driven selection of Performance Objectives, theoretically-grounded change methods and comprehensive pilot and review, IM provided the means by which to develop a retinal screening promotion intervention that was both evidence-based and sensitive to the needs and characteristics of young adults with T2D. However, despite this being a relatively simple, single-focus intervention, we shared the experience of other programmes, which reported the IM process to be both resource and time-intensive [28, 29, 79]. In particular, we found the high degree of process documentation time-consuming, although we acknowledge that this activity was crucial for transparency of reporting, and conforms to key items in the Template for Intervention Description and Replication (TIDieR) checklist and guidance [80].

### Strengths and limitations

The key strengths of this work relate to the use of IM, which combines both innovative and traditional intervention development activities into an organised, systematic process, and which is consistent with the UK MRC framework for the design and evaluation of complex interventions [24]. In the face of limited existing evidence, the empirical needs assessment, complemented by contribution from the multidisciplinary planning team, key stakeholders and the young adults with T2D PPI group, enabled comprehensive exploration of the problem, providing a robust foundation to the intervention. Further, the use of sound theoretical underpinnings, causal modelling, and detailed pilot and review, provided assurance as to the validity of the outcome. As such, the ‘Who is looking after your eyes?’ leaflet was both evidence-based and sensitive to the needs and characteristics of young adults with T2D.

Nonetheless, this study was subject to several limitations. First, the vast majority of studies targeting youth and young adults with T2D face recruitment challenges [20, 47, 81], and our empirical studies were no different in this respect. Despite numerous steps taken to improve recruitment, only 10 young adults with T2D participated in the qualitative study and only 2% of the eligible population completed the quantitative online study.

It is likely that recruitment was impacted by a range of challenges typically specific to young adults with T2D, such as social disadvantage, disengagement with existing services, and complex psychosocial and health needs [44, 46, 47, 82]. Furthermore, the needs assessment studies were conducted concurrent with a number of other research projects managed by the NDSS, which may have contributed to study ‘fatigue’ for this already small population (personal communication, D. Rae, National Inventory Manager, NDSS). Although low sample size potentially impacted the generalisability of the needs assessment findings, the response rate for the national survey was larger than any other conducted to date with this priority population.

Second, this study was limited to one priority population where in fact, several populations have been identified as at-risk for low retinal screening and vision loss from DR. These include young adults with T1D, those living in socio-economically deprived areas or from minority ethnic and Indigenous populations [83–86], each of which warrant targeted evidence-based intervention, informed by population-specific needs assessments.

Finally, many key contextual elements (e.g. intervention level, delivery medium and format) were externally prescribed within a broader sphere of real-world logistic and contractual limitations. Although unavoidable, this limitation meant that our intervention was unable to address external factors known to impact screening behaviour, such as the cultural diversity of young adults with T2D, low socioeconomic status and lack of English language proficiency, potentially limiting effectiveness. Given that NDSS database strictures limited the intervention to a format suited to postal delivery, the leaflet design was suited to the stated purpose for state-wide implementation. Diabetes Victoria has ensured sustainability and reach of the intervention by regularly updating their resources and making an electronic version of the leaflet freely available on its website [70].

### Future directions

Recent research suggests that an individual’s beliefs about diabetes and self-management, are most likely to be influenced early in their diabetes trajectory [87]. Certainly, this appears to be the case for retinal screening where, once initiated, the behaviour is generally sustained [73]. Thus, we recommend targeting individuals recently diagnosed with T2D via the NDSS, with registration date considered a proxy for date of diabetes diagnosis. The leaflet could be used to promote national retinal screening programmes in this age group and would be of greatest benefit if translated into additional languages. Further, this process could be utilised to produce tailored resources designed to increase awareness and screening for other populations at high-risk of DR (such as young adults with T1D), or for other diabetes-related complications which impact young adults with T2D (such as nephropathy and cardiovascular disease [88]).

Our experience of the time and resource-intensive nature of IM reinforces that expressed by others and we suggest that undertaking the full IM methodology may not be suitable for all situations. As such, we recommend that future programme planners explore alternative options where possible, such as adapting an existing, effective intervention to their target population. This can be enabled by use of a simplified process (IM Adapt), which guides decisions regarding selection of appropriate intervention, and components, to adapt [89].

## Conclusions

In conclusion, our mixed method needs assessment has highlighted salient challenges faced by young adults with T2D and we have demonstrated that IM is a feasible and worthwhile approach to use for the development of an evidence-based, engaging resource to promote retinal screening to young adults with T2D. This detailed illustration will enable researchers and health promotion specialists to adopt IM methods when developing interventions tailored to high-risk groups. Meanwhile, preliminary evaluation of the ‘Who is looking after your eyes?’ leaflet shows it meets the needs of young adults with T2D and its effectiveness in promoting uptake of retinal screening can now be evaluated in a fully-powered RCT.

## Additional files

Additional file 1: Literature study. Literature study procedure and findings. This file describes the procedure and findings of the literature study component of the needs assessment. (DOCX 78 kb)

Additional file 2: Interview guide. Interview guide used in qualitative component of needs assessment. This file presents all interview guide items which comprise the in-depth qualitative interview component of the needs assessment. (DOCX 42 kb)

Additional file 3: Modifiable behavioural determinants by baseline retinal screen (*N* = 129). This file presents individual items and findings from the quantitative online survey component of the needs assessment. (DOCX 22 kb)

Additional file 4: Matrix of Change Objectives. This file presents the complete matrix of Change Objectives (an illustrative example is provided in-text in Table 6). The Change Objectives are created at the intersection point of the five targeted modifiable behavioural determinants (Knowledge, Attitudes, Normative Beliefs, Intention, Behavioural Skills) in columns, and sub-objectives (from in-text Table 5), in rows. (DOCX 19 kb)

Additional file 5: Intervention map linking leaflet content directly back to Performance Objectives and Change Objectives. This file presents a complete intervention map (an illustrative example is provided in-text in Table 8). The intervention map links all leaflet content directly back to the Performance Objectives (specified in-text in Table 5) and the Change Objectives (Illustrated in-text in Table 6 and presented in full, in Additional file 4. (DOCX 37 kb)

## Abbreviations

DR: Diabetic retinopathy
GP: General practitioner
IM: Intervention mapping
MRC: Medical Research Council
NDSS: National Diabetes Services Scheme
SAM: Suitability assessment of materials
T2D: Type 2 diabetes mellitus
