# Supplementary material for: A tailored intervention to promote uptake of retinal screening among young adults with type 2 diabetes - an intervention mapping approach
Source: BMC Health Serv Res. 2018 May 31;18:396. doi: 10.1186/s12913-018-3188-5 (PMC5984467; doi:10.1186/s12913-018-3188-5)
Supplement: Supplementary file 1 — Literature study. Literature study procedure and findings. This file describes the procedure and findings of the literature study component of the needs assessment. (DOCX 78 kb) [file 12913_2018_3188_MOESM1_ESM.docx]

**Literature study procedure and findings**

**Literature study procedure**

We conducted a preliminary literature search (see box), which focused on screening facilitators and barriers, effective elements of existing individual-level screening interventions, and health information-seeking preferences and behaviours of the priority population. The literature study was maintained throughout the project via regular updates from search alerts; search terms were reviewed annually.

**Search engines and key databases:** Cochrane Reviews, EBSCOhost, ProQuest, Scopus, Psychlit, Medline, PubMed and CINAHL

**Publication period:** 10 years prior to February 2013.

**Search terms:** ‘young-onset’, ‘early-onset’, ‘young adult’, ‘type 2 diabet*’), in combination with terms associated with eye health and retinal screening uptake (‘barrier’, ‘challenge’, ‘facilitator’, ‘enabler’, ‘diabetic retinopathy’, ‘uptake’, ‘retinal screening‘, ‘eye health’, ‘ophthalmic’) and terms associated, more broadly, with screening and health behaviours of importance to young adults (e.g. ‘prevention’, ‘health promotion’, ‘colorectal cancer’, ‘cervical screening’, ‘breast screen, ‘sexual health’, ‘condoms’).

The search was supplemented by scanning reference lists of articles identified by our search strategy and grey literature (e.g. academic theses, government publications).

**Literature study findings**

***Screening facilitators and barriers***

Although barriers and facilitators to screening varied across countries, healthcare systems and population groups, shared themes were identified. For example, screening uptake depended on population awareness of health risk [1], health consequences [2] and treatment benefits following identification through screening [3]. Common awareness-related barriers included lack of awareness of DR, asymptomatic nature of early-stage DR, confusion between screening for DR and a standard vision-related eye check, absence of clinician recommendation and lack of diabetes self-management education [4-13]. Conversely, improvements in the above were common facilitators of screening [8, 14, 15]. In addition to lack of awareness, common screening barriers included low perceived personal risk; fear of screening or of a DR diagnosis if screening was undertaken, and denial of diabetes. Common screening facilitators included anticipated regret at not screening (e.g. undetected eye damage), clinician referral, social support from family, and social comparison [1, 6, 7, 10].

The evidence base specific to young adults with T2D was scant, with the majority of information either from pediatric-focused population-based studies or clinical trials [16, 17], or from retrospective medical record data [18, 19]. Younger age (under 40 years) was often controlled or excluded in adult-focused T2D and ophthalmic studies [20, 21], and the few available studies targeted specific ethnic or cultural groups, limiting generalisability [9, 22]. More broadly, the review identified modifiable factors impacting young adults’ health behaviours, including elevated rates of psychological distress [20, 23-25], perceptions of invulnerability leading to lowered perceived risk [26, 27], and disengagement with healthcare (including diabetes-specific) services [12, 27, 28]. Such factors are likely to have relevance for screening uptake among young adults with T2D. The need to target a range of behavioural determinants was reinforced by studies focusing specifically on populations at high risk of vision loss (e.g. low socio-economic status, suboptimal glycemic control), which singled out perception of personal risk [2], cultural appropriateness, and literacy levels of patient education materials [29], as important screening barriers and consequently, considerations for intervention.

***Effective elements of existing individual-level screening interventions***

A systematic review by Zhang of the effectiveness of interventions to promote retinal screening [30], reported that most interventions significantly increased screening rates, suggesting that a range of methodologies were effective. However, the lack of specificity of the effective elements of screening interventions [31], combined with lack of interventions specifically targeting young adults with T2D, highlight considerable gaps in the evidence base. Research suggests that screening interventions with the highest degree of effectiveness involve raising knowledge and awareness of DR, and/or involved ‘registration, reminder and recall’ (i.e. where a patient registered on a database received regular screening prompts) [30, 32]. The former (increasing knowledge and awareness) is an achievable intervention target in an Australian context. However, the reminder/recall is not because, currently, Australia does not have a nationally coordinated DR screening programme, such as that offered in the United Kingdom where people with diabetes are registered and automatically receive a screening invitation [33].

***Health information-seeking preferences and behaviours***

The information needs of people living with diabetes change over time dependent upon life stage and progress of the condition [34]. Young adults with T2D nominated their primary sources of diabetes-related information as: parents/family, healthcare practitioners, the national diabetes advocacy organisation, and the internet [35-37], with credibility of information given priority over delivery medium [28, 38].

Literature focusing more broadly on young adult health behaviours demonstrated that knowledge is a weaker correlate of preventative health behaviour compared to other modifiable factors, such as subjective norms, self-efficacy and risk perception [39-41]. This highlights the importance of including motivation and skills, as well as knowledge, as behaviour change targets for this group.

**References**

1. Strutton R, Du Chemin A, Stratton IM, and Forster AS. System-level and patient-level explanations for non-attendance at diabetic retinopathy screening in Sutton and Merton (London, UK): a qualitative analysis of a service evaluation. BMJ Open. 2016; 6(5):e010952.

2. MacLennan PA, McGwin G, Heckemeyer C, and et al. Eye care use among a high-risk diabetic population seen in a public hospital's clinics. JAMA Ophthalmology. 2014; 132(2):162-167.

3. Hua W, Cao S, Cui J, Maberley D, and Matsubara J. Analysis of reasons for noncompliance with laser treatment in patients of diabetic retinopathy. Canadian Journal of Ophthalmology / Journal Canadien d'Ophtalmologie. 2013; 48(2):88-92.

4. Klein R and Klein BEK. The epidemiology of eye disease: From glycemia to genetics: The Friedenwald lecture. Investigative Ophthalmology and Visual Science. 2006; 47(5):1747-1753.

5. Peng P-H, *Assessment of the factors associated with the acceptance of retinal screening among patients with diabetes in Taiwan*, in *Arnold School of Public Health*. 2010, University of South Carolina.

6. Ellish NJ, Royak-Schaler R, Passmore SR, and Higginbotham EJ. Knowledge, attitudes, and beliefs about dilated eye examinations among African-Americans. Ophthalmology and Vision Science. 2007; 48(5):1989-1994.

7. Hipwell AE, Sturt J, Lindenmeyer A, Stratton I, Gadsby R, O'Hare PO, et al. Attitudes, access and anguish: a qualitative interview study of staff and patients’ experiences of diabetic retinopathy screening. BMJ Open. 2014; 4(12):e005498.

8. John A, Cooper J, and Serrant-Green L. Barriers to diabetic retinopathy screening in south Asian groups. Primary Health Care. 2014; 24(8):25-30.

9. Al-Alawi A, Al-Hassan A, Chauhan D, Al-Futais M, and Khandekar R. Knowledge, attitude, and perception of barriers for eye care among diabetic persons registered at employee health department of a Tertiary Eye Hospital of Central Saudi Arabia. Middle East African Journal of Ophthalmology. 2016; 23(1):71-74.

10. Lewis K, Patel D, Yorston D, and Charteris D. A qualitative study in the United Kingdom of factors influencing attendance by patients with diabetes at ophthalmic outpatient clinics. Ophthalmic Epidemiology. 2007; 14(6):375-380.

11. Hartnett ME, Key IJ, Loyacano NM, Horswell RL, and DeSalvo KB. Perceived barriers to diabetic eye care: qualitative study of patients and physicians. Archives of ophthalmology. 2005; 123(3):387-391.

12. Müller A, Lamoureux E, Bullen C, and Keeffe JE. Factors associated with regular eye examinations in people with diabetes: Results from the Victorian Population Health Survey. Optometry and Vision Science. 2006; 83(2):96-101.

13. Livingston PM, Wood CA, McCarty CA, Harper CA, Keeffe JE, and Taylor HR. Awareness of diabetic retinopathy among people who attended a diabetic retinopathy screening program. Medical Journal of Australia. 1998; 169(2):117.

14. van Eijk KND, Blom JW, Gussekloo J, Polak BCP, and Groeneveld Y. Diabetic retinopathy screening in patients with diabetes mellitus in primary care: Incentives and barriers to screening attendance. Diabetes research and clinical practice. 2012; 96(1):10-16.

15. Walker EA, Basch CE, Howard CJ, Zybert PA, Kromholz WN, and Shamoon H. Incentives and barriers to retinopathy screening among African-Americans with diabetes. Journal of Diabetes and its Complications. 1997; 11(5):298-306.

16. TODAY study group. Retinopathy in Youth With Type 2 Diabetes Participating in the TODAY Clinical Trial. Diabetes Care. 2013; 36:3.

17. Hanman RF, Bell RA, Dabelea D, D'Agostino RB, Jr., Dolan L, Imperatore G, et al. The SEARCH for Diabetes in Youth Study: Rationale, Findings, and Future Directions. Diabetes care. 2014; 37(12):3336-3344.

18. Wong J, Molyneaux L, Constantino M, Twigg SM, and Yue DK. Timing is everything: age of onset influences long-term retinopathy risk in Type 2 diabetes, independent of traditional risk factors. Diabetes Care. 2008; 31(10):1985-1991.

19. Al-Saeed AH, Constantino MI, Molyneaux L, D’Souza M, Limacher-Gisler F, Luo C, et al. An inverse relationship between age of type 2 diabetes onset and complication risk and mortality: the impact of youth-onset type 2 diabetes. Diabetes Care. 2016; 39:823-829.

20. Hessler DM, Fisher L, Mullan JT, Glasgow RE, and Masharani U. Patient age: a neglected factor when considering disease management in adults with type 2 diabetes. Patient Education and Counseling. 2011; 85(2):154-159.

21. Forward H, Hewitt AW, and Mackey DA. Missing X and Y: a review of participant ages in population-based eye studies. Clinical & Experimental Ophthalmology. 2012; 40(3):305-319.

22. Hall CE, Hall AB, Kok G, Mallya J, and Courtright P. A needs assessment of people living with diabetes and diabetic retinopathy. BMC Research Notes. 2016; 9(1).

23. Anderson BJ, Edelstein S, Abramson NW, Levitt Katz LE, Yasuda PM, Lavietes SJ, et al. Depressive symptoms and quality of life in adolescents with type 2 diabetes: Baseline data from the TODAY study. Diabetes Care. 2011; 34(10):2205-2207.

24. Berge JM, Bauer KW, Eisenberg ME, Denny K, and Neumark-Sztainer D. Psychosocial and health behavior outcomes of young adults with asthma or diabetes. J Community Med Health Educ. 2013; 2(4):144-156.

25. Chittleborough CR, Winefield H, Gill TK, Koster C, and Taylor AW. Age differences in associations between psychological distress and chronic condition. International Journal of Public Health. 2011; 56:71-80.

26. Lapsley DK and Hill PL. Subjective invulnerability, optimism bias and adjustment in emerging adulthood. Journal of Youth and Adolescence. 2010; 39(8):847-857.

27. Nguyen TT, Jayadeva V, Cizza G, Brown RJ, Nandagopal R, Rodriguez LM, et al. Challenging recruitment of youth with type 2 diabetes into clinical trials. Journal of Adolescent Health. 2014; 54(3):247-254.

28. Savage S, Dabkowski S, and Dunning T. The education and information needs of young adults with type 2 diabetes: a qualitative study. Journal of Nursing & Healthcare of Chronic Illnesses. 2009; 1(4):321-330.

29. Elam AR and Lee PP. High-risk populations for vision loss and eye care underutilization: a review of the literature and ideas on moving forward. Survey of Ophthalmology. 2013; 58(4):348-358.

30. Zhang X, Norris SL, Saadine J, Chowdhury FM, Horsley T, Kanjilal S, et al. Effectiveness of interventions to promote screening for diabetic retinopathy. American journal of preventive medicine. 2007; 33(4):318-335.

31. Lawrenson JG, Graham-Rowe E, Lorencatto F, Burr J, Bunce C, Francis JJ, et al. Interventions to increase attendance for diabetic retinopathy screening. Cochrane Database of Systematic Reviews. 2018(1).

32. Halbert RJ, Kwan-Moon L, Nichol JM, and Legorreta AP. Effect of multiple patient reminders in improving diabetic retinoathy screening: A randomized trial. Diabetes Care. 1999; 22(5):752-755.

33. Scanlon PH. The English national screening programme for sight-threatening diabetic retinopathy. Journal of Medical Screening. 2008; 15(1):1-4.

34. Beeney LJ, Bakry AA, and Dunn SM. Patient psychological and information needs when the diagnosis is diabetes. Patient Education and Counseling. 1996; 29:109-116.

35. Diabetes Australia. Young Adults With Diabetes Needs Analysis. 2006 <http://static.diabetesaustralia.com.au/s/fileassets/diabetes-australia/6321f173-1642-42ed-8501-e95dc1ffa189.pdf>. Accessed: 22 August 2017.

36. Dunning T and Savage S. Information needs of young adults with type 2 diabetes: a literature review. Australian Diabetes Educator. 2013; 14:19-25.

37. Greene JA, Choudhry NK, Kilabuk E, and Shrank WH. Online social networking by patients with diabetes: a qualitative evaluation of communication with Facebook. J Gen Intern Med. 2011; 26(3):287-292.

38. Kumah-Crystal YA, Hood KK, Ho Y-X, Lybarger C, O'Connor BH, Rothman RL, et al. Technology use for diabetes problem solving in adolescents with type 1 diabetes: relationship to glycemic control. Diabetes Technology and Therapeutics. 2015; 17(7):449-454.

39. Bengel J, Belz-merk M, and Farin E. The role of risk perception and efficacy cognitions in the prediction of HIV-related preventive behavior and condom use. Psychology & Health. 1996; 11(4):505-525.

40. DiClemente RJ. Predictors of HIV-Preventive sexual behaviour in a high-risk adolescent population: the influence of perceived peer norms and sexual communication on incarcerated adolescents' consistent use of condoms. Journal of Adolescent Health. 1991; 12:385-390.

41. Abraham C, Krahe B, Dominic R, and Fritsche I. Do health promotion messages target cognitive and behavioural correlates of condom use? A content analysis of safer sex promotion leaflets in two countries. British Journal of Health Psychology. 2002; 7:227-233.
