# Supplementary material for: A tailored intervention to promote uptake of retinal screening among young adults with type 2 diabetes - an intervention mapping approach
Source: BMC Health Serv Res. 2018 May 31;18:396. doi: 10.1186/s12913-018-3188-5 (PMC5984467; doi:10.1186/s12913-018-3188-5)
Supplement: Supplementary file 2 — Interview guide. Interview guide used in qualitative component of needs assessment. This file presents all interview guide items which comprise the in-depth qualitative interview component of the needs assessment. (DOCX 42 kb) [file 12913_2018_3188_MOESM2_ESM.docx]

## Interview guide used in qualitative interview component of needs assessment

| **Warm up questions** |
| --- |

Q. Perhaps we can start with you telling me about what happened when you found out that you had type 2 diabetes.

(Prompts: were you initially advised to have eyes examined? By whom?

What did it mean to you? Did you do anything about it at the time? Did you follow advice?)

1. On a scale of 0 to 10, how much does your diabetes affect your life?

[Where 0 is ‘No affect at all’ and 10 is ‘Severely affects my life’ ]

0 1 2 3 4 5 6 7 8 9 10

No affect at all Severely affects my life

Can you please tell me why you nominated____________________________?

______________________________________________________________

Why did you pick x and not 0?_______________________________________

_______________________________________________________________

Why did you pick x and no 10? ______________________________________

_______________________________________________________________

2. On a scale of 0 to 10, where 0 is ‘Not at all confident’ and 10 is ‘Very confident’,

how confident are you that you can take care of your diabetes in the longer term?

0 1 2 3 4 5 6 7 8 9 10

Not at all confident Very confident

Can you please tell me why you nominated_____________________________?

3. Thinking about diabetes in the longer term, what is it that concerns you the most?

(Prompt: complications, greatest fear?) __________________________________________________________________

4. I am going to list 5 possible complications of diabetes.

Please rate in order of concern to you:

Nephropathy (kidney damage) Retinopathy (vision loss)

Heart disease Amputation (due to nerve damage) Stroke

What was your reason for choosing ________

as most concern?_______________________________________________________

What was your reason for choosing____ as least concern (ie: 5)?____________

**Interviewer:** I would like to focus the rest of the questions on one area of long term care, which is care of your eyes and your vision.

**Knowledge**

5. Are you aware of a connection between diabetes and eye health?

If Y: in your own words, can you please tell me what it is? _____________________

**Optimism**

6. Do you think that you are likely to experience vision problems due to diabetes?

Can you tell me why?________________________________

**Knowledge**

7. Have you previously heard of diabetic retinopathy?__________________________

If Y: in your own words, can you please tell me what it is? _____________________

[**Interviewer:** provide brief, simple explanation of retinopathy, if appropriate]

8. Do you know of anything that a person can do to reduce their risk of getting retinopathy,

or of slowing its progress?

If Y: in your own words, can you please tell me? __________________________

9. I am going to list 5 things that people do, to lower the risk or slow the progression of diabetic

retinopathy in the longer term.

Please rate in order of importance to you.

| Keeping HbA1c levels in target | Keeping blood pressure in target |
| --- | --- |
| Having regular eye examination | Keeping cholesterol at target |
| Keep a regular check on blood glucose levels | |

What was your reason for choosing___as most important (ie: 1)?___________

What was your reason for choosing__as least important (ie: 5)? ____________

[**Interviewer:** I would like to focus most of the rest of the questions on having regular eye examinations. These eye exams are usually done by putting drops in your eyes, which dilate your pupil. A photo is then taken of the inside of your eye, to look for damage to your retina. The rest of the questions will be about this kind of eye exam. Some of the questions may sound repetitive, but please answer all questions to help us best understand your point of view.]

**Beliefs about consequences**

10. What are the positive benefits to having eye exams?___________________________________

Are there are any negatives or ‘down sides’ to having eye exams?

Does one outweigh the other?

**Reinforcement**

11. (If previous eye exam) Did the experience of having an eye examination make it more or

less likely that you would have another one in the future? Why?___________________

**Beliefs about consequences**

12. What do you expect will happen immediately after having an eye examination?______

(Hypothetically) if you were diagnosed with DR, how do you think you would feel?

13. What do you expect will happen if you don’t have regular eye examinations?_________

**Knowledge**

14. Do you know the recommended target for blood glucose (HbA1c) to prevent complications

like retinopathy?

[**Interviewer:** HbA1c is an indicator of a patient‘s individual control over blood glucose levels in the past 90-120 days. [For patients with diabetes, the HbA1c target should be less than 7%. Every 1% reduction in HbA1c lowered the risk of DR by 30–40%]

15. Do you know the recommended target for blood pressure to prevent complications like

retinopathy?

[**Interviewer:** the target for systolic blood pressure should be less than 130 mmHg or 130/80

mmHg]

16. Do you know of any recommendations or guidelines that say how often people with

diabetes should have their eyes examined? If Y: please describe_____________________

**Skills, Knowledge**

17. Can you please describe how you would go about getting an eye examination? _______

**Social/Professional Role and Identity**

18. Are you comfortable with people knowing that you have type 2 diabetes?

How did people react when you told them? Is there anyone you wouldn’t want to know that

you have diabetes?

19. What does having eye examinations mean to you?

(Prompt: what kind of person are they?)

20. Do you belong to any diabetes support groups or forums?

*(Prompt for full list)*

**Social Influence**

21. Have you been prompted by someone to have an eye examination?___________

(Prompt: for full list)

What was your response?

22. If anyone could influence your decision to have an eye examination, who would it be?

(Prompt: for full list)

Why would their views influence you?

23. Has anyone you know had an eye examination for DR? How did that make you feel?

24. Do you know of anyone who has experienced vision problems or lost their vision because of diabetes or diabetic retinopathy? How does that make you feel?

**Knowledge**

25. What steps would you take if you did notice changes in your vision (spots or blurred vision)?

**Goals**

26. Considering your other priorities, on a scale of 0 to 10, with 0 being ‘not at all important’ 10

being ‘very important’, how important is it for you to maintain your current vision?

0 1 2 3 4 5 6 7 8 9 10

Not at all important Very important

(If not 10) What are higher priorities and why?

**Intentions**

27. Considering your other competing priorities, on a scale of 0 to 10, with 0 being ‘not at all

likely’, and 10 being ‘extremely likely’, how likely is it for you to have regular eye

examinations when they are next due?

0 1 2 3 4 5 6 7 8 9 10

Not at all likely Extremely likely

(If not 10) What are higher priorities and why?___

[**Interviewer**: the major benefit of regular eye examinations is early detection of DR before people experience symptoms of vision loss. Once DR is detected, treatment to slow the progression of the condition can begin.]

28. Now that I have explained the benefits of eye examinations, on a scale of 0 to10, where 0 is

‘not at all likely’ and 10 is ‘extremely likely’, how likely are you to have an eye examination

when it is next due?

0 1 2 3 4 5 6 7 8 9 10

Not at all likely Extremely likely

(Prompt: If the two values are different, explore)

**Beliefs about capabilities**

29. One a scale of 0 –10, where 0 is ‘not at all confident’ and 10 is ‘very confident’, how

confident are you that you can talk to your GP or diabetes educator about eye

examinations?

0 1 2 3 4 5 6 7 8 9 10

Not at all confident Very confident

What makes it easy/hard? What do you think would help you to overcome these problems?

**Emotion**

30. Can you please imagine/think back to, receiving a reminder for your regular eye

examination, what feelings would you/did you have?

31. Can you please imagine/think back to when you are having an eye examination, what

thoughts or feelings would you/did you, have?

32. Please imagine the time immediately after you have an eye examination, what thoughts or

feelings do you have? _______________________________________________________

33. One a scale of 0 –10, where 0 is ‘not at all concerned’ and 10 is ‘extremely concerned’, do

thoughts of vision loss worry or concern you?

0 1 2 3 4 5 6 7 8 9 10

Not at all concerned Extremely concerned

(Interviewer to explore)

**Behavioural regulation**

34. Eye examinations don’t have to be done as often as other diabetes self-management tasks,

such as taking medication or checking blood glucose. If you want to have an eye

examination, how do you think you will remember (or remind yourself)?

(Prompt: for detail)_____________

**Memory, attention and decision processes**

35. Have you ever forgotten, or delayed, an eye examination when it was due?

If Y: do you know why? (Prompt for detail)

36. Making the decision to have an eye exam:

**If previously had an eye exam:** Please step me through how you made the decision to have

an eye examination?

**If not previously had eye exam:** Did you consciously decide against going for an eye exam?

What went through your mind when you made that decision?

(Prompt: Please step me through thought process)

**Environmental context and resources**

37. Sometimes our plans are hindered by things outside of our control.

What things, outside of your control, could make it harder for you to have regular eye

exams? What things could make it easier? (Prompt: list)

38. How/where do you get information regarding diabetes management?

(Prompt: What have you found useful? Preferred format? Suggest improvements)

39 How/where do you get information regarding eye examinations and diabetic eye conditions?

(Prompt: What have you found useful? Preferred format? Suggest improvements).

**Closing**

Thank you very much for taking part in our study. Is there anything else about this topic that you would like to mention?
