# Supplementary material for: A tailored intervention to promote uptake of retinal screening among young adults with type 2 diabetes - an intervention mapping approach
Source: BMC Health Serv Res. 2018 May 31;18:396. doi: 10.1186/s12913-018-3188-5 (PMC5984467; doi:10.1186/s12913-018-3188-5)
Supplement: Supplementary file 3 — Modifiable behavioural determinants by baseline retinal screen (N = 129). This file presents individual items and findings from the quantitative online survey component of the needs assessment. (DOCX 22 kb) [file 12913_2018_3188_MOESM3_ESM.docx]

**Additional file 3:** Modifiable behavioural determinants by baseline retinal screen (N=129)

| **Modifiable behavioural determinants** | **Retinal screen** | | ***p-value*** |
| --- | --- | --- | --- |
|  | **No (n=33)** | **Yes (n=96)** |  |
| **INFORMATION (KNOWLEDGE) ITEMS** | | | |
| **Diabetes can lead to vision loss** | 30 (91) | 93 (97) | .174 |
| **All people with diabetes are at risk of DR** | 26 (79) | 89 (93) | .004 |
| **Approximately 1 in 4 people with T2D have DR** | 8 (24) | 38 (40) | .164 |
| **DR can cause vision loss or blindness** | 29 (91) | 92 (99) | .051 |
| **DR can develop without symptoms** | 15 (47) | 56 (61) | .268 |
| **DR is influenced by high blood pressure** | 10 (31) | 44 (47) | .167 |
| **DR is influenced by high cholesterol** | 7 (22) | 29 (31) | *.*437 |
| **DR is treatable if detected early via an eye health**  **check** | 21 (66) | 65 (70) | .819 |
| **DR is more likely to develop the longer you have diabetes** | 21 (66) | 68 (73) | .561 |
| **DR is influenced by high blood glucose** | 23 (72) | 83 (89) | .025 |
| **Early DR is asymptomatic** | 4 (12) | 9 (9) | .738 |
| **Recommended target HbA1c^a^** | 17 (53) | 81 (87) | <.001 |
| **Recommended target blood pressure** | 21 (66) | 67 (72) | .644 |
| **Optometrist most likely to conduct DR examination** | 31 (94) | 89 (96) | .652 |
| **Initiate eye examinations ‘at diabetes diagnosis’** | 5 (15) | 42 (45) | .004 |
| **Screen ‘at least every 2 years’ if no DR present** | 0 (0) | 18 (19) | .003 |
|  | | | |
| **MOTIVATION ITEMS^b^** | | | |
| **Attitudes to retinal screening^c^:**  **An eye health check for DR would be...** | | | |
| **...a good idea** | 4.19 (1.14) | 4.94 (0.23) | .001 |
| **...(not) ‘unpleasant’** | 3.71 (0.94) | 3.86 (1.07) | .500 |
| **...wise** | 4.29 (0.97) | 4.86 (0.44) | .004 |
| **...(not) ‘difficult’** | 3.10 (1.17) | 4.02 (0.96) | <.001 |
| **...(not) ‘frightening’** | 3.52 (1.06) | 4.07 (1.13) | .019 |
| **...(not) ‘unnecessary’** | 3.52 (1.18) | 4.50 (0.62) | <.001 |
| **...reassuring** | 3.94 (0.96) | 4.63 (0.61) | <.001 |
| **...important** | 4.06 (1.06) | 4.89 (0.35) | <.001 |
| **...beneficial** | 4.03 (0.95) | 4.86 (0.38) | <.001 |
| **...comfortable** | 3.26 (1.15) | 3.68 (1.10) | .073 |
| **...empowering** | 3.10 (1.19) | 3.73 (0.97) | .004 |
| **11 items (range: 11-55, α=.86)** | 40.71 (8.42) | 48.03 (4.26) | <.001 |
|  | | | |
|  | | | |
| **MOTIVATION ITEMS^b^ (Cont.)**  **Attitudes: risk perception^d^** | | | |
| **I believe I will develop DR due to my diabetes** | 4.03 (1.45) | 4.14 (1.62) | .734 |
| **I expect to be diagnosed with DR at my next eye**  **health check** | 2.97 (1.47) | 2.43 (1.66) | .114 |
| **I believe I can reduce my risk of vision problems**  **if I manage my diabetes well** | 2.32 (1.44) | 1.43 (0.79) | .002 |
| **I believe I will develop vision problems due to**  **diabetes** | 4.26 (1.69) | 4.49 (1.68) | .511 |
| **4 items (range: 4-28, α=.70)** | 13.58 (4.46) | 12.50 (4.34) | .238 |
|  | | | |
| **Attitudes: anticipated regret^d^**  **If I did NOT have an eye health check for DR, I would feel…** | | | |
| **...indifferent** | 3.65 (1.64) | 3.24 (1.89) | .295 |
| **...concerned** | 5.03 (1.70) | 5.88 (1.40) | .007 |
| **...fearful** | 4.48 (1.79) | 5.13 (1.70) | .073 |
| **...worried** | 4.65 (1.80) | 5.53 (1.47) | .007 |
| **...regretful** | 4.81 (1.68) | 5.54 (1.49) | .023 |
| **...guilty** | 4.58 (1.75) | 5.47 (1.57) | .010 |
| **6 items (range: 6-42, α=.87)** | 22.61 (7.02) | 25.33 (6.04) | .040 |
|  | | | |
| **Normative beliefs^d^**   1. **Subjective norms** | | | |
| **My family/close friends would approve of me**  **attending an eye health check** | 5.94 (1.69) | 6.82 (0.80) | .008 |
| **My health professionals would approve of me**  **attending an eye health check** | 5.84 (1.75) | 6.94 (0.28) | .001 |
| **2 items (range: 2-14; α=.93, r=.87)** | 11.77 (3.43) | 13.77 (1.02) | .003 |
| 1. **Descriptive norms** | | | |
| **Most people I know with diabetes have regular**  **eye health checks** | 4.55 (1.57) | 5.07 (1.42) | .091 |
|  | | | |
| **Intention^d^** | | | |
| **I plan to attend an eye health check…** | 4.26 (2.32) | 6.76 (0.77) | <.001 |
| **I will make an effort to have an eye health check…** | 4.55 (2.42) | 6.74 (0.87) | <.001 |
| **I intend to have an eye health check…** | 4.42 (2.32) | 6.74 (0.77) | <.001 |
| **3 items (range: 3-21, α=.98)** | 13.22 (6.97) | 20.24 (2.16) | <.001 |
|  | | | |
| **BEHAVIOURAL SKILLS ITEMS^b,e^** | | | |
| **Perceived control**  **How confident are you that you...** | | | |
| **…know what steps you can take to reduce the risk**  **of developing DR?** | 2.39 (1.17) | 3.06 (1.29) | .012 |
| **…will have regular eye health checks?** | 3.16 (1.16) | 4.43 (0.87) | <.001 |
| **…know how to make the appointment for an eye**  **check?** | 3.35 (1.36) | 4.54 (0.83) | <.001 |
| **…will remember to have an eye health check in**  **the next four weeks OR when it is next due?** | 2.68 (1.35) | 4.36 (0.90) | <.001 |
| **…will attend the eye health check that you have**  **booked?** | 3.84 (1.16) | 4.67 (0.60) | <.001 |
| **…can reschedule the eye health check to a**  **different time or day if needed? (n=119)** | 3.45 (1.15) | 4.61 (0.67) | <.001 |
| **6 items (range: 6-30, α=.87)** | 18.87 (5.44) | 25.63 (3.75) | <.001 |
|  | | | |
| **Overcoming barriers**  **How confident are you that you…** | | | |
| **…can talk to your doctor about your eye health?** | 3.39 (1.28) | 4.17 (1.03) | .001 |
| **…can find the time to attend an eye health check**  **in the next four weeks OR when it is next due?** | 2.74 (1.37) | 4.55 (0.75) | <.001 |
| **…will mention you have diabetes when you make**  **the eye check appointment?** | 3.68 (1.25) | 4.65 (0.62) | <.001 |
| **…can resume your normal activities immediately**  **after the eye health check?** | 3.55 (1.26) | 3.91 (1.25) | .169 |
| **…can afford to pay for the eye health check, if**  **there is a charge?** | 2.68 (1.49) | 3.52 (1.48) | .008 |
| **5 items (range: 5-25, α=.76)** | 16.03 (4.88) | 20.80 (3.49) | <.001 |

DR: diabetic retinopathy. Data are number (%) of participants who answered each item correctly (Knowledge items); mean (SD) Motivation and Behavioural skills items.

*p-value* is Pearson’s Chi-Square (or Fisher’s exact test if expected cell count<5), or Independent-samples t-test (two-sided); statistical significance *p*<0.01.

^a^Glycated haemoglobin (measure of average blood glucose over the past 8-12 weeks, and indicator of DR risk).

^b^Minimal missing data (average 6%, range 2-11%). Valid n: 121 (motivation items), 120 (behavioural skills items, unless noted otherwise). Cronbach’s alpha noted (plus Pearson’s r for 2-item constructs). Some items responses reverse coded where required.

Item response range: ^c^1 (Strongly disagree) to 5 (Strongly agree), ^d^1 (Strongly disagree) to 7 (Strongly agree), ^e^1 (Not at all confident) to 5 (Extremely confident)
