# Supplementary material for: A tailored intervention to promote uptake of retinal screening among young adults with type 2 diabetes - an intervention mapping approach
Source: BMC Health Serv Res. 2018 May 31;18:396. doi: 10.1186/s12913-018-3188-5 (PMC5984467; doi:10.1186/s12913-018-3188-5)
Supplement: Supplementary file 4 — Matrix of Change Objectives. This file presents the complete matrix of Change Objectives (an illustrative example is provided in-text in Table 6). The Change Objectives are created at the intersection point of the five targeted modifiable behavioural determinants (Knowledge, Attitudes, Normative Beliefs, Intention, Behavioural Skills) in columns, and sub-objectives (from in-text Table 5), in rows. (DOCX 19 kb) [file 12913_2018_3188_MOESM4_ESM.docx]

**Matrix of Change Objectives**

| **Performance Objectives and sub-objectives^** | **Modifiable behavioural determinants** | | | | | | | | | |
| --- | --- | --- | --- | --- | --- | --- | --- | --- | --- | --- |
|  | **Knowledge** | | **Attitudes** | | **Normative beliefs** | | **Intention** | | **Behavioural skills** | |
| **PO.1 Young adults with type 2 diabetes will demonstrate a clear understanding of diabetic retinopathy (DR)** | | | | | | | | | | |
| 1. **Modifiable and non-modifiable DR risk factors** | **K.1.1** Understand DR and know key modifiable risk factors | |  | |  | | **I.1.1** Form a positive intention to actively manage modifiable DR risk factors | | **BS.1.1** Identify and initiate the actions required to reduce risk of developing DR | |
| **Clinical targets**  **for reducing**  **risk of DR** | **K.1.2** Know clinical targets for modifiable DR risk factors to prevent DR or slow progression | |  | |  | |  | | **BS.1.2** Believe that they can avoid negative consequences | |
| 1. **Symptoms of DR** | **K.1.3** Understand asymptomatic nature of early DR and explain symptoms | |  | |  | |  | |  | |
| **Role of DR in**  **vision loss** | **K.1.4** Understand how DR affects the eye | | **A.1.4** Perceive consequences for family unit/future family | |  | |  | |  | |
| 1. **Prevalence of DR** | **K.1.5** Know that DR is a common complication of diabetes | |  | | **NB.1.5** Believe that similar others are at risk of DR | |  | |  | |
| **PO.2 Young adults with type 2 diabetes will demonstrate a clear understanding of retinal screening** | | | | | | | | | | |
| **Role in**  **detecting DR**  **and reducing**  **vision loss** | | **K.2.1** Know the role of retinal screening in reducing vision loss | | **A.2.1** Explain the clinical benefit of retinal screening | |  | |  | |  |
| 1. **Screening procedure and experience** | | **K.2.2** Know when to have first and subsequent retinal screen | | **A.2.2** Believe that screening promotes positive feelings | | **NB.2.2** Believe that similar others approve of, and would recommend screening | |  | | **BS.2.2** Express confidence in retinal screening procedure (prepare reader for the experience) |
| 1. **Booking and examination procedure** | | **K.2.3** Know that retinal screening can be self-referred | |  | |  | | **I.2.3** Form an intention to book first screen soon | | **BS.2.3** Be confident that they can get an eye health check |
| **PO.3 Young adults with type 2 diabetes will be motivated to engage in retinal screening** | | | | | | | | | | |
| **Prioritise retinal**  **screening** | |  | |  | | **NB.3.1** Recognise that similar others have overcome screening barriers | | **I.3.1** Form an intention to prioritise retinal screening | |  |
| 1. **Understand personal risk of DR** | | **K.3.2** Know that DR risk increases over time | | **A.3.2** Perceive high personal risk and susceptibility to DR | |  | |  | |  |
| **PO.3 Young adults with type 2 diabetes will be motivated to engage in retinal screening (Cont.)** | | | | | | | | | | |
| 1. **Identify personal barriers to retinal screening** | |  | | **A.3.3** Believe that attending screening will relieve fear and guilt and be a positive experience | | **NB.3.3** See that similar others face screening barriers (e.g. cost, fear of adverse effects) | |  | | **BS.3.3** Be confident in one’s ability to identify and overcome common screening barriers |
| 1. **Perceive personal responsibility to engage in screening** | | **K.3.4** Know that they can take steps to protect eye health | | **A.3.4** Adopt personal responsibility for retinal screening | | **NB.3.4** Believe that similar others take responsibility for their own eye health | |  | | **BS.3.4** Be confident they have the tools to act on personal responsibility |
| **PO.4 Young adults with type 2 diabetes will proactively engage with the healthcare system and their healthcare team** | | | | | | | | | | |
| **Discuss diabetes**  **and eye health**  **with healthcare**  **professionals** | | **K.4.1a** Know that GP should be involved in monitoring diabetes-related eye health  **K.4.1b** Know that an eye examination for DR is different to a standard eye check | | **A.4.1** Anticipate a positive social and emotional experience | | **NB.4.1** Believe that similar others approve of, and recommend, sharing their diabetes diagnosis with optometrist | |  | | **BS.4.1a** Prompt GP contact  **BS.4.1b** Be confident in sharing diabetes diagnosis with optometrist |
| **PO.4 Young adults with type 2 diabetes will proactively engage with the healthcare system and their healthcare team (Cont.)** | | | | | | | | | | |
| 1. **Understand treatment benefits and options** | | **K.4.2** Know treatment trajectory | | **A.4.2** Understand benefits of early treatment | |  | |  | | **BS.4.2** Know that they will receive expert advice |
| **Seek more**  **information about**  **diabetes and eye**  **health** | | **K.4.3a** Know how to find more information (e.g. optometrist, diabetes or DR)  **K.4.3b** Know that information is available in other languages | |  | |  | | **I.4.3** Form intention to access credible information about DR and screening | |  |

PO=Performance Objective, DR=diabetic retinopathy, GP=general practitioner.

Determinants: K=Knowledge, A=Attitudes, NB=Normative Beliefs, I=Intention, BS=Behavioural Skills

^See Table 5 for full list of Performance Objectives and sub-objectives
