# Supplementary material for: A tailored intervention to promote uptake of retinal screening among young adults with type 2 diabetes - an intervention mapping approach
Source: BMC Health Serv Res. 2018 May 31;18:396. doi: 10.1186/s12913-018-3188-5 (PMC5984467; doi:10.1186/s12913-018-3188-5)
Supplement: Supplementary file 5 — Intervention map linking leaflet content directly back to Performance Objectives and Change Objectives. This file presents a complete intervention map (an illustrative example is provided in-text in Table 8). The intervention map links all leaflet content directly back to the Performance Objectives (specified in-text in Table 5) and the Change Objectives (Illustrated in-text in Table 6 and presented in full, in Additional file 4. (DOCX 37 kb) [file 12913_2018_3188_MOESM5_ESM.docx]

**Intervention map linking leaflet content directly back to Performance Objectives and Change Objectives**

| **Performance Objectives (PO) and Change Objectives^** | | | | | | | **Leaflet content (antecedent leaflet text in brackets illustrates context)** | | **Panel No.*** |
| --- | --- | --- | --- | --- | --- | --- | --- | --- | --- |
| **PO.1** | | | | **YOUNG ADULTS WITH T2D WILL DEMONSTRATE A CLEAR UNDERSTANDING OF DIABETIC RETINOPATHY (DR)** | | | | | |
| **PO.1.1** | | | | **Modifiable and non-modifiable DR risk factors** | | | | | |
|  | **K.1.1** Understand DR and know key modifiable risk factors | | | | | **What is diabetes eye disease?**  Diabetes eye disease is also called Diabetic Retinopathy (DR).  It is caused by having high blood glucose levels over a long time. Other things that increase your risk of DR are high blood pressure and high cholesterol. | | | 4 |
|  | **I.1.1** Form a positive intention to actively manage modifiable DR risk factors | | | | | (What can I do to protect myself from DR and prevent vision loss?)  3. Follow your diabetes treatment plan which includes the diabetes ABCs | | | 5 |
|  | **BS.1.1** Identify and initiate the actions required to reduce risk of developing DR | | | | | (What happens if I have DR?)   - You **can** slow progression of DR by keeping your blood glucose, blood pressure and cholesterol as close to target as possible. | | | 8 |
| **PO.1.2** | | | | **Clinical targets for reducing risk of DR** | | | | | |
|  | **K.1.2** Know clinical targets for modifiable DR risk factors to prevent DR or slow progression | | | | | | | **A** Average blood glucose (HbA1c) below 7% (53mmol/mol)  Every 1% (11mmol/mol) decrease in HbA1c lowers your risk of developing DR  by 30–40%.  **B** Blood pressure below 130/80 mm Hg  Keeping your blood pressure at target slows progression of DR  **C** Cholesterol at target  LDL cholesterol less than 2.0 mmol/L, triglycerides less than 2.0 mmol/L) | 5 |
| **PO.1** | | | | **YOUNG ADULTS WITH T2D WILL DEMONSTRATE A CLEAR UNDERSTANDING OF DR (Cont.)** | | | | | |
| **PO.1.2** | | | | **Clinical targets for reducing risk of DR (Cont.)** | | | | | |
|  | **BS.1.2** Believe that they can avoid negative consequences | | | | | | | (The **good news** is) there are things you can do to reduce your risk  (The **good news** is) this leaflet provides the information you need to help **prevent** vision loss from DR. | 1  4 |
| **PO.1.3** | | | | **Symptoms of DR** | | | | |  |
|  | **K.1.3** Understand asymptomatic nature of early DR and explain symptoms | | | | | | | **Will I know if I have DR?**  You may not know. In the early stages, DR **has no symptoms at all**. In the later stages, you my notice blurred, hazy or double vision or you may have sudden loss of vision. | 4 |
| **PO.1.4** | | | | **Role of DR in vision loss** | | | | | |
|  | **K.1.4** Understand how DR affects the eye | | | | | (What is diabetes eye disease?) DR damages the tiny blood vessels in the back of your eye. If left untreated, your vision can be affected.  (Jane 25 years, diagnosed with type 2 diabetes 3 years ago) "You might have good vision, you might think that your eyes are absolutely brilliant and there's no issue. But in the back of your eye, there could be a problem with those little tiny veins that you don't realise." | | | 4  4 |
| **PO.1** | | | | **YOUNG ADULTS WITH T2D WILL DEMONSTRATE A CLEAR UNDERSTANDING OF DR (Cont.)** | | | | | |
| **PO.1.4** | | | | **Role of DR in vision loss (Cont)** | | | | | |
|  | **A.1.4** Perceive consequences for family unit / future family | | | | | Image: mother and daughter smiling. Child holding hands over mother’s eyes  (Lucas, aged 34, diagnosed with type 2 diabetes 2 years ago) "I'm a busy person and my family depend on me. “I know I can’t do all the things I do without my sight."  (Jenny’s story: before and after the eye health check: “I was scared. I was scared of what damage was done…of confronting the fact that my eyesight could be damaged, and of going through the exam and being confronted with what’s there.”)  “But I want to take care of my kids; I want to be able to see their children one day. I do want to be able to grow older and have my vision.” | | | 1  1  7 |
| **PO.1.5** | | | | **Prevalence of DR** | | | | | |
|  | **K.1.5** Know that  DR is a common complication of diabetes | | | | There are over 34,000 Australians with type 2 diabetes who are under 40 years of age. More than 8,500 will already have DR. | | | | 1 |
|  | **NB.1.5** Believe that similar others are  at risk of DR | | | | **But I'm still young. Am I at risk of DR?**  Yes you are. **Anyone** with diabetes can develop DR, which is the leading cause of vision loss for people under 60 years  (Jane, 25 years, diagnosed with type 2 diabetes 3 years ago) "You might have good vision, you might think that your eyes are absolutely brilliant and there's no issue. But in the back of your eye, there could be a problem with those little tiny veins that you don't realise." | | | | 1  4 |
| **PO.2** | | | **YOUNG ADULTS WITH T2D WILL DEMONSTRATE A CLEAR UNDERSTANDING OF RETINAL SCREENING (Cont.)** | | | | | | |
| **PO.2.1** | | | **Role in detecting DR and reducing vision loss** | | | | | | |
|  | | **K.2.1** Know the role  of retinal screening in reducing vision loss | | | | | - Having a diabetes eye health check and treating DR early can prevent severe vision   loss. | | 1 |
|  | | **A.2.1** Explain the clinical benefit of retinal screening | | | | | The only way to know if you have DR is to have a diabetes eye health check | | 4 |
| **PO.2.2** | | | **Screening procedure and experience** | | | | | | |
|  | | **K.2.2** Know when to have first and subsequent retinal screen | | | | | Have a diabetes eye health check when diabetes is first diagnosed and then at least  every 2 years (more often if recommended by your optometrist) | | 5 |
|  | | **A.2.2** Believe that screening promotes positive feelings | | | | | (Jenny’s story: before and after the eye health check) “Overall, it was worth it and the thought that I can control this gives me real peace of mind.” | | 7 |
|  | | **NB.2.2** Believe that similar others approve of, and would recommend, screening | | | | | (Jenny's advice to you) "I suppose if I was telling someone that's just been diagnosed,  I would be saying to them 'Don't wait to be told and don't wait until you notice  changes - book an eye health check now’." | | 8 |
| **PO.2** | | | **YOUNG ADULTS WITH T2D WILL DEMONSTRATE A CLEAR UNDERSTANDING OF RETINAL SCREENING (Cont.)** | | | | | | |
| **PO.2.2** | | | **Screening procedure and experience (Cont.)** | | | | | | |
|  | | **BS.2.2** Express  confidence in retinal screening procedure (prepare reader for  the experience) | | | | | (What is a diabetes eye health check?)   - It is usually done by an optometrist who will take a photo of the back of your eye. - Your optometrist will look at the photo to check the blood vessels at the back of your   eye for signs of diabetes-related eye damage | | 6 |
| **PO.2.3** | | | **Booking and examination procedure** | | | | | | |
|  | | **K.2.3** Know that  retinal screening can be self-referred | | | | | (Getting a diabetes eye health check is easy)   - You don’t need a referral from your GP. You can book an appointment directly with   an optometrist. | | 6 |
|  | | **I.2.3** Form an  intention to book first screen soon | | | | | Book a diabetes eye health check now  Image: calendar | | 2 |
|  | | **BS.2.3** Be confident that they can get an eye health check | | | | | Getting a diabetes eye health check is easy. | | 6 |
| **PO.3** | | | **YOUNG ADULTS WITH T2D WILL BE MOTIVATED TO ENGAGE IN RETINAL SCREENING** | | | | | | |
| **PO.3.1** | | | **Prioritise retinal screening** | | | | | | |
|  | | **NB.3.1** Recognise that similar others have overcome screening barriers | | | | | (Jenny’s story: before and after the eye health check) “I was scared. I was scared of  what damage was done…of confronting the fact that my eyesight could be damaged,  and of going through the exam and being confronted with what’s there.” | | 7 |
| **PO.3** | | | **YOUNG ADULTS WITH T2D WILL BE MOTIVATED TO ENGAGE IN RETINAL SCREENING (Cont.)** | | | | | | |
| **PO.3.1** | | | **Prioritise retinal screening (Cont.)** | | | | | | |
|  | | **I.3.1** Form an  intention to prioritise  retinal screening | | | | | (What can I do to protect myself from DR and prevention vision loss?)  1. Have a diabetes eye health check.  (Note: eye health check listed as Step 1, highest priority) | | 5 |
| **PO.3.2** | | | **Understand personal risk of DR** | | | | | | |
|  | | **K.3.2** Know that DR risk increases over time | | | | | - The longer you have diabetes the more at risk you are of DR. | | 1 |
|  | | **A.3.2** Perceive high personal risk and susceptibility to DR | | | | | Image: mother and daughter smiling. Child holding hands over mother’s eyes  But I'm still young. Am I at risk of DR?  Yes you are. **Anyone** with diabetes can develop DR, which is the leading cause of  vision loss for people under 60 years.  There are over 34,000 Australians with type 2 diabetes who are under 40 years of age.  More than 8,500 will already have DR.   - The longer you have diabetes the more at risk you are of DR.   (Lucas, aged 34, diagnosed with type 2 diabetes 2 years ago)  "I didn't know that I was at risk."  (Jane 25 years, diagnosed with type 2 diabetes 3 years ago) "You might have good  vision, you might think that your eyes are absolutely brilliant and there's no issue.  But in the back of your eye, there could be a problem with those little tiny veins that  you don't realise." | | 1  1  1  1  1  4 |
| **PO.3** | | | **YOUNG ADULTS WITH T2D WILL BE MOTIVATED TO ENGAGE IN RETINAL SCREENING (Cont.)** | | | | | | |
| **PO.3.3** | | | **Identify personal barriers to retinal screening** | | | | | | |
|  | | **A.3.3** Believe that attending screening will relieve fear and guilt and be a positive experience | | | | | (Jenny’s story: before and after the eye health check) “It was actually quite fun; I  don’t know why I put it off. I was really scared going in there, but definitely not now –  I’m not fazed by it at all.” | | 7 |
|  | | **NB.3.3** See that similar others face screening barriers (e.g. cost, fear of adverse effects) | | | | | (Jenny’s story: before and after the eye health check) “The eye drops were a bit  uncomfortable and there was a small cost – but I think it’s a wise spend considering  what you’re preventing.” | | 7 |
|  | | **BS.3.3** Be confident in one’s ability to identify and overcome common screening barriers | | | | | (What else do I need to know?)   - A diabetes eye health check takes about 30 minutes.   (What else do I need to know?)  It may be free (bulk-billed) or there may be a small fee.  (What else do I need to know?)  Your optometrist may use eye drops which helps them to see the back of your eye.  If you do have eye drops, they may be a little uncomfortable. The drops will also leave  you sensitive to light, so bring your sunglasses and be prepared to wait a while for  your vision to return to normal | | 6  6  6 |
| **PO.3.4** | | | **Perceive personal responsibility to engage in screening** | | | | | | |
|  | | **K.3.4** Know that they can take steps to protect eye health | | | | | What can I do to protect myself from DR and prevent vision loss? | | 5 |
| **PO.3** | | | **YOUNG ADULTS WITH T2D WILL BE MOTIVATED TO ENGAGE IN RETINAL SCREENING (Cont.)** | | | | | |  |
| **PO.3.4** | | | **Perceive personal responsibility to engage in screening (Cont.)** | | | | | |  |
|  | | **A.3.4** Adopt personal responsibility for retinal screening | | | | | (Lucas, aged 34, diagnosed with type 2 diabetes 2 years ago)  "I'm a busy person and my family depend on me.”  Leaflet heading: Who is looking after your eyes? | | 1  3 |
|  | | **NB.3.4** Believe that similar others take responsibility for their own eye health | | | | | Images: mother and daughter, smiling couple selfie, young man  of indeterminate cultural origin, Asian female (a.k.a. ‘Jenny’) | | 1,3,5,8 |
|  | | **BS.3.4** Be confident they have the tools to  act on personal responsibility | | | | | Leaflet sub-heading: Your guide to preventing vision loss from diabetes eye disease  Protect your sight for life | | 3  2 |
| **PO.4** | | | **YOUNG ADULTS WITH T2D WILL PROACTIVELY ENGAGE WITH THE HEALTHCARE SYSTEM AND THEIR HEALTHCARE TEAM** | | | | | | |
| **PO.4.1** | | | **Discuss diabetes and eye health with your healthcare professionals** | | | | | | |
|  | | **K.4.1a** Know that GP should be involved in monitoring diabetes-related eye health | | | | | Either way, discuss your results with your GP or your diabetes specialist | | 6 |
|  | | **K.4.1b** Know that an eye examination for DR is different to a standard eye check | | | | | (What is a diabetes eye health check?)   - It is different to a standard eye check because it specifically looks to see whether   diabetes is affecting your eyes. | | 6 |
| **PO.4** | | | **YOUNG ADULTS WITH T2D WILL PROACTIVELY ENGAGE WITH THE HEALTHCARE SYSTEM AND THEIR HEALTHCARE TEAM (Cont.)** | | | | | | |
| **PO.4.1** | | | **Discuss diabetes and eye health with your healthcare professionals (Cont.)** | | | | | | |
|  | | **A.4.1** Anticipate a positive social and emotional experience | | | | | (Jenny’s advice to you)  "I had a lovely optometrist and she really put me at ease." | | 8 |
|  | | **NB.4.1** Believe that similar others approve of, and recommend, sharing their diabetes diagnosis with optometrist | | | | | (Jenny’s advice to you)  "Discuss with the optometrist what to expect, what you should be aware of and so  on.” | | 8 |
|  | | **BS.4.1a** Prompt GP contact | | | | | Either way, discuss your results with your GP or your diabetes specialist | | 6 |
|  | | **BS.4.1b** Be confident in sharing diabetes diagnosis with optometrist | | | | | (Getting a diabetes eye health check is easy. You don’t need a referral from your GP.  You can book an appointment directly with an optometrist.)  When you do, be sure to tell them you have diabetes. | | 6 |
| **PO.4** | | | | **YOUNG ADULTS WITH T2D WILL PROACTIVELY ENGAGE WITH THE HEALTHCARE SYSTEM AND THEIR HEALTHCARE TEAM (Cont.)** | | | | | |
| **PO.4.2** | | | | **Understand treatment benefits and options** | | | | | |
|  | | **K.4.2** Know treatment trajectory | | | | | What happens next?   - If they see any signs of damage to the back of your eye, your optometrist will either   monitor it or arrange treatment with an ophthalmologist (medical eye specialist).  (What happens if I have DR?)   - In the early stages, treatment may not be needed, but you may be asked to have eye   health checks more frequently to monitor the DR.  (What happens if I have DR?)   - If DR progresses, you may need to take tablets or have specialist treatment (usually   laser therapy) | | 6  8  8 |
|  | | **A.4.2** Understand benefits of early treatment | | | | | 2. Treat DR early  Early treatment can prevent up to 98% of severe vision loss | | 5 |
|  | | **BS.4.2** Know that they will receive expert advice | | | | | What happens if I have DR?   - Your eye health professional will advise you of your treatment options | | 8 |
| **PO.4** | | | **YOUNG ADULTS WITH T2D WILL PROACTIVELY ENGAGE WITH THE HEALTHCARE SYSTEM AND THEIR HEALTHCARE TEAM (Cont.)** | | | | | | |
| **PO.4.3** | | | **Seek more information about diabetes and eye health** | | | | | | |
|  | | **K.4.3a** Know how to find more information  (e.g. optometrist, diabetes or DR) | | | | | **To find an optometrist in your area**   - Scan the QR code to download the free Diabetes Australia app - Visit Optometry Australia   www.optometry.org.au/find-an-optometrist/  **For more information on eye health and diabetes management**   - Visit **diabetesvic.org.au**, or call the Info line on **1300 136 588** - Multilingual infoline 1300 801 164 **multiculturalportal.ndss.com.au** | | 2  2 |
|  | | **K.4.3b** Know that information is available in other languages | | | | | Image: national interpreter symbol | | 2 |
|  | | **I.4.3** Form intention  to access credible information about DR and screening | | | | | Images: smart phone, QR codes  ‘Proudly sponsored by [stakeholder logos]’ | | 2  2 |

PO=Performance Objective, DR=diabetic retinopathy, GP=general practitioner. *Panel number in *Figure 3.*

^See Table 5 for full list of Performance Objectives and sub-objectives; see Additional file 4 for complete list of Change Objectives.

K=Knowledge, A=Attitudes, NB-Normative Behaviour, I=Intention, BS=Behavioural Skills
